# Intracellular dynamics of Ataxin-2 in the human brains with normal and frontotemporal lobar degeneration with TDP-43 inclusions

**DOI:** 10.1186/s40478-020-01055-9

**Published:** 2020-10-28

**Authors:** Ryohei Watanabe, Shinji Higashi, Takashi Nonaka, Ito Kawakami, Kenichi Oshima, Kazuhiro Niizato, Haruhiko Akiyama, Mari Yoshida, Masato Hasegawa, Tetsuaki Arai

**Affiliations:** 1 Dementia Research Project, Tokyo Metropolitan Institute of Medical Science, 2-1-6 Kamikitazawa, Setagaya, Tokyo Japan; 2 Department of Psychiatry, University of Tsukuba, 1-1-1 Tennodai, Tsukuba, Ibaraki Japan; 3 Department of Psychiatry, Tokyo Metropolitan Matsuzawa Hospital, 2-1-1 Kamikitazawa, Setagaya, Tokyo Japan; 4 Department of Psychiatry, Tokyo Medical University Ibaraki Medical Center, 3-20-1 Chuo, Ami, Inashiki, Ibaraki Japan; 5 Brain Bank for Aging Research, Department of Neuropathology, Tokyo Metropolitan Institute of Gerontology, 35-2 Sakaecho, Itabashi, Tokyo Japan; 6 Department of Clinical Research, Yokohama Brain and Spine Center, 1-2-1 Takigashira, Isogo, Yokohama, Kanagawa Japan; 7 Institute for Medical Science of Aging, Aichi Medical University, 1-1 Yazakokarimata, Nagakute, Aichi Japan

**Keywords:** RNA-binding protein, Ataxin-2, Localization, TDP-43, Proteinopathy, Neurodegeneration, Amyotrophic lateral sclerosis, Frontotemporal lobar degeneration

## Abstract

TAR DNA-binding protein of 43 kDa (TDP-43) is a major component of intracellular aggregates formed in brains of the patients with frontotemporal lobar degeneration (FTLD) and amyotrophic lateral sclerosis (ALS), which are correctively referred to as TDP-43 proteinopathies. A link between Ataxin-2 (ATXN2) and TDP-43 proteinopathies was established when intermediate CAG repeat expansions of *ATXN2* gene were found to be associated with ALS and it was shown that ATXN2 modifies TDP-43 toxicity. Although ATXN2’s contribution to TDP-43 proteinopathies has been mostly studied in ALS, recent studies have shown that intermediate repeat expansions of *ATXN2* also influence the phenotype of FTLD by an unknown mechanism. To address this issue, we immunohistochemically and biochemically analyzed the intracellular dynamics of ATXN2 in brains of normal controls and FTLD-TDP cases. The immunohistochemical studies revealed that ATXN2 localized in the neuronal cytoplasm and proximal dendrites, and expressed widely and uniformly in normal human brains. A semi-quantitative immunofluorescent analysis of normal brains revealed that the cytoplasmic ATXN2 strongly associates with ribosomal protein S6 and poly-A binding protein 1 and partially overlaps with the endoplasmic reticulum marker Calnexin, suggesting a major role of ATXN2 in protein synthesis. The results of immunohistochemical and biochemical analyses of brains from FTLD-TDP cases showed the colocalization of ATXN2 and phosphorylated TDP-43 in the dystrophic neurites and the neuronal cytoplasmic inclusions in the hippocampal region, and a significant reduction of ATXN2 protein compared to controls. These results suggest that ATXN2 is involved in the pathological process of FTLD-TDP. It remains to be clarified whether reduced ATXN2 expression induces neurodegeneration by impairing protein synthesis or plays a neuroprotective role by attenuating the toxicity of TDP-43 aggregates in FTLD-TDP and other TDP-43 proteinopathies.

**Electronic supplementary material:**

The online version of this article (10.1186/s40478-020-01055-9) contains supplementary material, which is available to authorized users.

## Introduction

Amyotrophic lateral sclerosis (ALS) is an adult-onset motor neuron disease (MND), which is characterized by rapidly progressive muscle weakness and paralysis, resulting in death due to respiratory failure within 2–4 years [7]. Frontotemporal lobar degeneration (FTLD) is one of the presenile dementias which exhibit behavioral disturbances and language problems [38]. The affected brain regions sometimes overlap between the two diseases, corresponding with their clinical overlapping. In the brains of the patients with either disease, abnormal intracellular structures consisting of misfolded proteins such as the TAR DNA-binding protein of 43 kDa (TDP-43) and fused-in-sarcoma (FUS) are seen in the surviving neurons and glial cells in affected regions [1, 24, 29]. Furthermore, some pathogenic gene mutations are known to cause both ALS and FTLD phenotypes [34]. From these overlapping features, the concept of ALS-FTLD as a clinical-pathological-genetic continuum has been propounded and currently accepted [40].

TDP-43 is one of the major proteins that induce neurodegeneration by its intracellular accumulation in ALS-FTLD and other neurodegenerative diseases, which are collectively referred to as TDP-43 proteinopathies [1, 27, 29]. TDP-43 is one of the heterogenous nuclear ribonucleoproteins (hnRNPs), and ubiquitously distributes in many human organs, including the brain. It localizes mainly within the nucleus and functions in transcription and translation via binding to RNA and thus contributes to regulating protein expression [27]. In the remaining neurons of affected regions in ALS-FTLD patient’s brain, TDP-43 is markedly lost from the nucleus and mislocalizes to the cytoplasm. TDP-43 is subject to post-translational modifications such as fragmentation, hyper-phosphorylation, and ubiquitination, becomes insoluble, and then aggregates in the part of nucleus, cytoplasm, and neurites. These TDP-43 positive aggregates are referred to as neuronal intranuclear inclusions (NIIs), neuronal cytoplasmic inclusions (NCIs), and dystrophic neurites (DNs), respectively. FTLD-TDP is currently classified into four pathological subtypes based on the morphological aspects and distribution of these aggregates [21]. Type A has both NCIs and DNs mainly in II lamina of the neocortex, type B has numerous NCIs in all cortical layers, type C is characterized by predominant DNs in upper cortical layers with few NCIs except for those in the dentate gyrus and type D has many DNs and NIIs in all cortical layers. Such aggregates of TDP-43 convert nearby normal TDP-43 protein into the abnormal form which further propagates among cells [30]. The toxicity induced by these aggregates or the loss of function of TDP-43 is thought to induce neurodegeneration.

Intermediate repeat expansions (27-33 CAG repeats) in the exon 1 of the Ataxin-2 (ATXN2) gene is known as a genetic risk factor or phenotypic modifier in ALS [11, 26]. The pathogenic role of *ATXN2* in human disease was first reported in familial spinocerebellar ataxia type 2 (SCA2) cases, in which the mutant allele of *ATXN2* harboring highly expanded CAG/CAA repeats (> 34) was found [17, 18, 20]. Although the contribution of *ATXN2* to TDP-43 proteinopathies has been studied mostly in ALS, recent studies reported the pathogenic role of *ATXN2* in FTLD as a phenotypic modifier. Rubino et al. reported the association between *ATXN2* intermediate repeat expansions and an earlier age at onset, parkinsonism and psychotic symptoms in the initial phase of FTLD [36]. Lattante et al. reported that *ATXN2* intermediate repeat expansions are a strong risk factor not only in ALS but also in FTLD-ALS, and could act as a strong modifier of the FTLD phenotype in the presence of *C9orf72* repeat expansion [25]. Fournier et al. reported an autopsy proven case of FTLD-TDP with *ATXN2* intermediate repeat expansions and without pathogenic variants in known FTLD genes [13]. ATXN2 harboring an intermediately expanded polyglutamine (polyQ) tract expressed from mutant *ATXN2* is reported to promote abnormal modification of TDP-43 [15]. The suppression of normal endogenous ATXN2 alleviates TDP-43 accumulation [5]. These findings suggest that ATXN2 is involved in the pathogenesis of both ALS and FTLD.

Thus, ATXN2 is a unique protein in that its different polyQ expansions are associated with two different proteinopathies, polyQ disease and TDP-43 proteinopathies. It remains unknown why vulnerable brain regions differ depending on the length of polyQ expansions in ATXN2. While the pathological involvement of mutant ATXN2 with a highly expanded polyQ tract in SCA2 has been intensively investigated, the association between ATXN2 with a normal or intermediately expanded polyQ tract and the pathogenesis of TDP-43 proteinopathies is largely unknown.

The aim of this study is to clarify the role of ATXN2 in the pathogenesis of FTLD-TDP. To elucidate this issue, it seems important to investigate whether changes of expression or intracellular localization of ATXN2 occur in the brains of FTLD-TDP cases, since intracellular mislocalization or depletion of other TDP-43-interacting RBPs (such as FUS, hnRNPA1, and hnRNPA2/B1) has been found in brains of patients with ALS and multisystem proteinopathies and are considered to be involved in neurodegeneration [22, 28, 41]. Therefore, in this study, we examined the intracellular dynamics of ATXN2 in human brains of normal controls and FTLD-TDP cases using sensitive immunohistochemistry and western blotting. We also performed polysome profiling of SH-SY5Y cell lines and mouse brains to clarify the intracellular localization of ATXN2 in detail. The results showed a strong association of ATXN2 and ribosome in human brains, colocalization of ATXN2 to phosphorylated TDP-43 (pTDP-43)-positive aggregates, and the reduced expression of ATXN2 in brains of FTLD-TDP cases. These suggest a pathological involvement of ATXN2 in FTLD-TDP by impairing protein synthesis machinery or a neuroprotective role by attenuating the toxicity of TDP-43 aggregates.

## Materials and methods

### Human brain tissue

For immunohistochemical and biochemical analysis, postmortem human brain tissues were obtained from the Tokyo Metropolitan Matsuzawa Hospital and Aichi Medical University. Tissue samples were also supplied by the Manchester Brain Bank, which is part of the Brains for Dementia Research programme, jointly funded by Alzheimer’s Research UK and the Alzheimer’s Society. The cases’ profiles are shown in Table 1.

**Table 1:** Patients examined in this study

Case no.	Age at death (years)	Sex	Brain weight (g)	Postmortem delay (h)	Brain regions	Status of samples	Neuropathological diagnosis
1	72	M	1350	6	F, T, Hip, BG, midbrain	PFA	Cerebral infarct
2	71	M	1180	35	F, T, Hip	PFA, frozen brain	Normal control
3	77	F	1145	9	F, T, Hip	PFA, frozen brain	Normal control
4	72	M	N/A	3.3	F	Frozen brain	Cerebral embolism
5	75	M	1295	24	F	Frozen brain	Cerebral infarct
6	81	M	1240	4.5	F	Frozen brain	Normal control
7	79	M	N/A	3.35	F	Frozen brain	Normal control
8	79	M	N/A	N/A	T	FA	Normal control
9	75	M	1280	6.57	Cerebellum	PFA	Normal control
10	69	F	1145	5	BG, midbrain	PFA	Normal control
11	68	F	1200	37	BG	PFA	Normal control
12	68	M	1220	7	BG	PFA	Normal control
13	73	M	1400	N/A	Midbrain	PFA	Normal control
14	68	F	1160	24	SC	PFA	Normal control
15	68	M	N/A	N/A	T, Hip	PFA	FTLD-TDP type C
16	66	M	N/A	N/A	T, Hip	PFA, frozen brain	FTLD-TDP type C
17	81	M	950	N/A	T, Hip	PFA	FTLD-TDP type C
18	59	M	N/A	N/A	T	Frozen brain	FTLD-TDP type C
19	83	M	1415	19.8	T	Frozen brain	FTLD-TDP type C
20	75	M	1174	N/A	F	Frozen brain	FTLD-TDP type C
21	66	F	1035	N/A	F	Frozen brain	FTLD-TDP type C
22	71	F	955	N/A	F	Frozen brain	FTLD-TDP type A

*F* frontal cortex, *T* temporal cortex, *Hip* hippocampal region, *FA* formaldehyde-fixed brain sections, *PFA* paraformaldehyde-fixed brain sections, *FTLD* frontotemporal lobar degeneration, *BG* basal ganglia, *SC* spinal cord

### Mouse

A male 6-week-old mouse (individual recognition No. C57BL/6JJmsSlc; born on 22 July 2019 and bred at the Animal Research Division, Tokyo Metropolitan Institute of Medical Science) was used for this experiment.

### Construction of Halo-ATXN2 and Flag-ATXN2 vector

The full-length Halo-ATXN2 expression construct (pFN21A-Halo-ATXN2) was obtained (Promega, WI, USA). We also prepared the pcDNA3-Flag-ATXN2 construct by subcloning using PrimeSTAR Max DNA Polymerase and In-Fusion HD Cloning Kit (TaKaRa bio, Shiga, Japan).

### Maintenance and transfection of cell line

Human neuroblastoma SH-SY5Y cells (ATCC, MD, USA) were cultured in Dulbecco’s modified Eagle’s medium (DMEM)/F12 medium (Sigma-Aldrich, Darmstadt, Germany) supplemented with 10% (v/v) fetal calf serum, penicillin–streptomycin–glutamine (Gibco, CA, USA), and MEM nonessential amino acids solution (Gibco, CA, USA). The cells were maintained at 37 °C under a humidified atmosphere of 5% (v/v) CO_2_ in air. They were grown to 50% confluence in six-well culture dishes for transient expression and then transfected with expression plasmids (usually 1 μg) using X-tremeGENE 9 (Roche, Bazel, Switzerland) or FuGENE6 (Roche, Bazel, Switzerland) according to the manufacturer’s instructions.

### Preparation of sarkosyl-soluble fraction from transfected cultured cells and western blotting

Transfected SH-SY5Y cells were collected and washed with phosphate-buffered saline (PBS). Cells were lysed in 1 ml of A68 buffer (10 mM Tris–HCl, pH 7.4, 0.8 M NaCl, 1 mM EGTA, 5 mM EDTA, and 10% (w/v) sucrose) containing 1% (w/v) sarkosyl by sonication. After incubation at 37 °C for 30 min, cell lysates were ultracentrifuged at 150,000×*g* for 20 min at 25 °C. The supernatants were removed and collected as sarkosyl-soluble fractions. The protein concentrations of those fractions were determined with a Pierce BCA Protein Assay Kit (Thermo Fisher, MA, USA). Then, those fractions were added to SDS-sample buffer and boiled for 5 min. Western blotting was performed with mouse monoclonal anti-ATXN2 antibody (1:1000) and rabbit polyclonal anti-ATXN2 antibody (1:2000) as described in Table 2. The intensity of bands was detected by LAS-4000 luminescent image analyzer (Fujifilm, Tokyo, Japan).

**Table 2:** Primary antibodies used in this study

Primary antibodies	Type	Source	Dilution
Human Ataxin-2, 713-904	Mouse, monoclonal	BD biosciences (611378)	1:1000 (IPL), 1:500 (IF), 1:250–1000 (WB)
Human Ataxin-2, 1293-1313	Rabbit, polyclonal	Thermofisher (#PA5-78845)	1:2000 (IF), 1:2000 (WB)
Human ribosomal protein S6 (5G10)	Rabbit, monoclonal	CST (#2217)	1:500 (IF), 1:3000 (WB)
Human PolyA-binding protein 1	Rabbit, polyclonal	CST (#4992)	1:500 (IF)
Human Calnexin (C5C9)	Rabbit, monoclonal	CST (#2679)	1:500 (IF)
Human Lysosome associated membrane protein 1	Rabbit, polyclonal	Abcam (ab24170)	1:1000 (IF)
Human Golgi Glycoprotein 1	Rabbit, polyclonal	Abcam (ab103439)	1:500 (IF)
Human TDP-43 (2E2-D3)	Mouse, monoclonal	Abnova (H00023435-M01)	1:500 (IF)
Phosphorylated TDP-43 (pS409/410)	Rabbit, polyclonal	Made by Dr. M. Hasegawa [16]	1:1000 (WB)
Anti-Glyceraldehyde-3-Phosphate Dehydrogenase Antibody (6C5)	Mouse, monoclonal	Chemicon (MAB374)	1:3000 (WB)

*IPL* immunoperoxidase labeling, *IF* immunofluorescence, *WB* western blotting

### Immunohistochemistry and confocal microscopy of human brain

For neuropathological examinations, small blocks of human brain tissues were dissected at autopsy and fixed in 4% (w/v) paraformaldehyde (PFA) (cases 1, 2, 3, 9, 10, 11, 12, 13, 14, 15, 16, and 17 in Table 1) or 10% (w/v) formaldehyde (FA) (case 8) in PBS for 72 h. PFA-fixed brain slices were transferred to 20% (w/v) sucrose in PBS, cut into 30 µm sections on a freezing microtome (Leica, Wetzlar, Germany), and stored at 4 °C in 20% (w/v) sucrose in PBS with 0.1% (w/v) sodium azide. Sections were mounted onto FRONTIER coating glass slides (Matsunami, Osaka, Japan) and were allowed to dry. Formalin-fixed brain tissues were embedded in a paraffin wax block, cut into 10 µm sections using a microtome (Yamato Kohki, Saitama, Japan), and mounted as above. Sections were pretreated by autoclaving for 20 min in 10 mM sodium citrate buffer, pH 6.0, at 121 °C or by pressure cooking (Groupe SEB, Écully, France) for 10 min in 10 mM Tris base, 1 mM EDTA solution buffer, pH 9.0, at 120 °C as previously described with minor modification [23]. Sections were incubated with 1.5% (v/v) H_2_O_2_ in 70% (v/v) methanol for 30 min to inactivate endogenous peroxidases, blocked with 5% (v/v) normal goat serum in PBS with 0.3% (v/v) Triton X-100 for 1 h, and incubated for 3 days with the appropriate primary antibody cocktail. The antibodies and dilution concentrations in this study are summarized in Table 2.

For immunoperoxidase labeling of normal controls (cases 1, 2, 3, 8, 9, 10, 11, 12, 13, and 14) and FTLD-TDP cases (cases 15, 16, and 17), after incubating with a cocktail containing either of two anti-ATXN2 antibodies: a monoclonal (611378; BD Biosciences) or a polyclonal (#PA5-78845; Thermofisher), the sections were incubated with a biotinylated secondary antibody for 2 h, detected by the avidin-biotinylated horseradish peroxidase complex (ABC) method (Vectastain Elite Kit, Vector Laboratories, CA, USA), and visualized with diaminobenzidine (Wako chemical, Tokyo, Japan). Sections were counterstained with hematoxylin.

For single- or double-immunofluorescence labeling of normal controls (cases 1, 2, and 3) and FTLD-TDP cases (cases 15, 16, and 17), brain sections were pretreated and incubated for 3 days with a cocktail containing one or two of the following primary antibodies: two anti-ATXN2 antibodies as mentioned above, anti-PolyA-binding protein 1 (PABP1) antibody (polyclonal, #4992, CST), anti-TDP-43 antibody (monoclonal, H00023435-M01, Avnova), several organelle markers including endoplasmic reticulum (ER) marker (anti-Calnexin, monoclonal, #2679, CST), ribosomal marker (anti-ribosomal protein S6 (RPS6), monoclonal, #2217, CST), lysosome marker (anti-Lysosome associated membrane protein 1 (LAMP1), polyclonal, ab24170, abcam), and Golgi apparatus marker (anti-Golgi Glycoprotein 1 (GLG1), polyclonal, ab103439, abcam). The sections were washed and incubated with cocktail of FITC-conjugated goat anti-mouse IgG (Proteintech, IL, USA) and/or TRITC-conjugated goat anti-rabbit IgG (Proteintech, IL, USA). The sections were post-treated with TrueBlack (Biotium, CA, USA) to reduce autofluorescence. After further washing, the sections were coverslipped with ProLong Diamond Antifade Mountant with DAPI (Thermofisher, MA, USA). Fluorescent images with a resolution of 241 nm were acquired as two serial z-stacks of 2 μm intervals using a TCS-SP8 confocal microscope (Leica, Wetzlar, Germany) and LAS X software (Leica, Wetzlar, Germany).

### Quantitative analysis of fluorescent images

The obtained fluorescent images from three controls (case 1, 2, and 3) and three FTLD-TDP cases (case 15, 16, and 17) were processed and analyzed using Fiji software (National Institutes of Health, MD, USA).

For the semi-quantitative intensity analysis of cytoplasmic molecules, PFA-fixed brain sections of the six cases were immunostained by the same procedure. The integrated density and corrected total cell fluorescence (CTCF) within the automatically determined fifty cytoplasmic regions-of-interest (ROIs) was calculated as previously described [33]. The integrated density represents the mean fluorescent intensity within ROIs per unit area (μm^2^). CTCF represents the corrected value of integrated density after background subtraction and was calculated based on the formula (Integrated density − (Area of ROI × mean background fluorescence)). The mean integrated density and CTCF values obtained from both groups were plotted and statistically compared.

For the colocalization analysis of ATXN2 and cytoplasmic organelle markers or the related molecules, PABP1 and TDP-43, double-immunofluorescent images obtained from the six cases were subjected to background subtraction and 2D deconvolution. Then, Pearson’s correlation coefficients above threshold (PCC) and Manders’ colocalization coefficients above threshold (tM) 1 and 2 (tM_1_ and tM_2_) were calculated. PCC measures the overall linear relationship from − 1 (perfectly inversely related) to 1 (perfectly related). A value greater than − 0.5 and less than 0.5 is taken to mean that the two probes are unrelated. The formula for PCC from a Green and Red color image is given as follows:

$$ {\text{PCC}} = \frac{{\Sigma _{i} \left( {R_{i} - \bar{R}} \right) \times \left( {G_{i} - \bar{G}} \right)}}{{\sqrt {\Sigma _{i} \left( {R_{i} - \bar{R}} \right)^{2} \times\Sigma _{i} \left( {G_{i} - \bar{G}} \right)^{2} } }} $$

where *G*_*i*_ and *R*_*i*_ refer to the intensity values of the Green and Red channels, respectively, of pixel *i,* and $\bar{G}$ and $\bar{R}$ refer to the mean intensities of the Green and Red channels, respectively, across the entire image. Of two tM values, tM_1_ shows the fraction of the Green signal in compartments containing the Red signal above the automatically determined threshold ranging from 0 to 1. Similarly, tM_2_ shows the fraction of the Red signal in compartments containing the Green signal above threshold. There is no shared threshold value for tM as to the deemed significant overlap or non-overlap. For convenience, we decided a tentative classification for tM values as high (0.8 ≤ tM), moderate (0.65 ≤ tM < 0.8), and low (tM < 0.65). The formula for tM_1_ and tM_2_ is given as follows:

$$ {\text{tM}}_{1} = \frac{{\Sigma _{i} G_{{i,{\text{colocal}}}} }}{{\Sigma _{i} G_{i} }}  \;{\text{and}} \;{\text{tM}}_{2} = \frac{{\Sigma _{i} R_{{i,{\text{colocal}}}} }}{{\Sigma _{i} R_{i} }} $$

where *G*_*i*,collocal_ = *G*_*i*_ if *R*_*i*_> calculated threshold and *G*_*i*,collocal_ = 0 if *R*_*i*_ ≤ calculated threshold, and *R*_*i*,collocal_ = *R*_*i*_ if *G*_*i*_> calculated threshold and *R*_*i*,collocal_ = 0 if *G*_*i*_ ≤ calculated threshold. tM_1_ and tM_2_ was calculated for the colocalization analysis using the above-mentioned monoclonal and polyclonal ATXN2 antibodies, respectively. Each measured coefficient value from at least fifteen cytoplasmic ROIs was plotted. The result of colocalization analysis was also graphically represented in two-dimensional scatterplots where the intensity of green color (x-axis) is plotted against the intensity of red color (y-axis) for each pixel.

### Polysome profiling of cultured cells and mouse brain

The polysome profiles from the SH-SY5Y cell line and mouse brain were analyzed as described with minor modifications [32, 39]. SH-SY5Y cells were grown in four 10 cm dishes to about 90% confluence. Then, cells were cultured in medium with 0.1 mg/ml cycloheximide (Wako chemical, Tokyo, Japan) at 37 °C for 15 min, washed twice with ice-cold PBS containing 0.1 mg/ml cycloheximide, and then lysed in 1 ml of polysome lysis buffer (25 mM Tris–HCl, pH 7.5, 100 mM KCl, 5 mM MgCl_2_, 0.2 mg/ml heparin, 0.3% (w/v) Triton X-100, and 1 mM dithiothreitol). The cell suspension was transferred to a microfuge tube and incubated on ice for 10 min with occasional inverting every 2 min. Post-mitochondrial supernatants were obtained by centrifugation at 10,000×*g* for 10 min at 4 °C. The supernatant (0.9 ml) was transferred to a fresh tube and carefully loaded onto 10 ml of 10–50% (w/v) continuous linear sucrose gradient in polysome lysis buffer. The gradient was ultracentrifuged at 259,000×*g* for 90 min at 4 °C using swing rotors. Each gradient was collected into 0.75 ml fractions from the bottom of the tube with continuous absorbance monitoring at 260 nm. For protein precipitation, each fraction was incubated on ice with the same volume of 20% (v/v) trichloroacetic acid solution for 30 min. After centrifugation at 15,000×*g* for 10 min at 4 °C, the supernatant was discarded, and the pellet was washed with ice-cold acetone and centrifuged again at 15,000×*g* for 10 min at 4 °C. The supernatant was removed and allowed to dry. The dried pellet was dissolved in 80 μl of PBS containing 1% (w/v) SDS by sonication and boiled for 5 min. Equal volumes were used for Western blotting using mouse monoclonal anti-ATXN2 antibody (1:250) and rabbit monoclonal RPS6 antibody (1:3000) (Table 2).

For the polysome profiling of mouse brain, a 6-week-old mouse was sacrificed by pentobarbital anesthesia and decapitation. The brain was removed and placed in 4 ml per brain ice-cold polysome lysis buffer as mentioned above. Brain tissue was immediately homogenized with 12 strokes in a motor-driven glass-Teflon homogenizer and placed on ice. The homogenized tissue was transferred to a fresh tube and centrifuged at 10,000×*g* for 20 min at 4 °C. The supernatant was loaded onto a sucrose gradient. From this point, the lysate was processed as were the SH-SY5Y cells. For EDTA disruption of polyribosomes, 25 mM EDTA was used in the place of MgCl_2_ in polysome lysis buffer.

### Quantitative western blotting analysis of human brain

For the quantitative western blotting analysis of human brain, a RIPA-soluble fraction, particulate fraction, and sarkosyl-insoluble fraction were prepared as previously described with minor modification [6, 10, 17]. Frozen brain tissue (0.2 g) was homogenized in 10 volumes (v/w) of RIPA buffer (50 mM Tris–HCl, pH 8.0, 150 mM NaCl, 1 mM EDTA, 1 mM EGTA, 0.5% (w/v) sodium deoxycholate, 0.1% (w/v) SDS, 1 mM PMSF, and a tablet of protease inhibitor cocktail (Roche, Bazel, Switzerland)) containing 1% (w/v) Nonidet P-40 in a motor-driven glass-Teflon homogenizer. The lysate was incubated on ice for 15 min and centrifuged at 16,000 xg for 20 min at 4 °C. A portion of each supernatant was added to SDS-sample buffer and boiled for 5 min (RIPA-soluble fraction). The residual 900 μl supernatant was transferred into a fresh tube and further centrifuged at 150,000×*g* for 20 min at 4 °C. The supernatants were removed, and the pellets were solubilized in SDS-sample buffer and boiled for 5 min (particulate fraction). The pellet of the preceding 16,000×*g* centrifugation was solubilized in 20 volumes (v/w) of A68 buffer containing 2% (w/v) sarkosyl, incubated for 30 min at 37 °C, and then centrifuged at 15,000×*g* for 10 min at 25 °C. The supernatant was transferred into a fresh tube and was further centrifuged at 150,000×*g* for 20 min at 25 °C. The pellets were solubilized in SDS-sample buffer and boiled for 5 min (sarkosyl-insoluble fraction). The protein concentration of each RIPA-soluble fraction was determined with a Pierce BCA Protein Assay Kit (Thermo Fisher, MA, USA). Western blotting of the equal loading mass of each sample was detected with mouse monoclonal anti-ATXN2 antibody (1:500), rabbit monoclonal RPS6 antibody (1:3000), and rabbit polyclonal phosphorylated TDP-43 (pTDP-43) antibody (1:1000) (Table 2). Glyceraldehyde-3-phosphate dehydrogenase (GAPDH) was also detected to ensure the reliability of data. The signal intensity of each band was analyzed and quantified using Fiji software (National Institutes of Health, MD, USA). The mean intensity values obtained from six controls (case 2, 3, 4, 5, 6, and 7) and six FTLD-TDP cases (case 16, 18, 19, 20, 21, and 22) were plotted and statistically compared.

### Statistical analysis

Unpaired Student T tests were used to determine the significance in the quantitative intensity analysis of fluorescent images. The level of significance was set at *P *≤ 0.05 (*P ≤ 0.05 and ns = *P* > 0.05). The Mann–Whitney U tests were used to determine significance in the quantitative western blotting analysis of human brain. The level of significance was set at *P *< 0.01 (*P < 0.01 and ns = *P* ≥ 0.01). Plotted data are mean ± standard deviation throughout.

## Results

### ATXN2 is widely distributed in human brain neurons

**Fig. 1:**
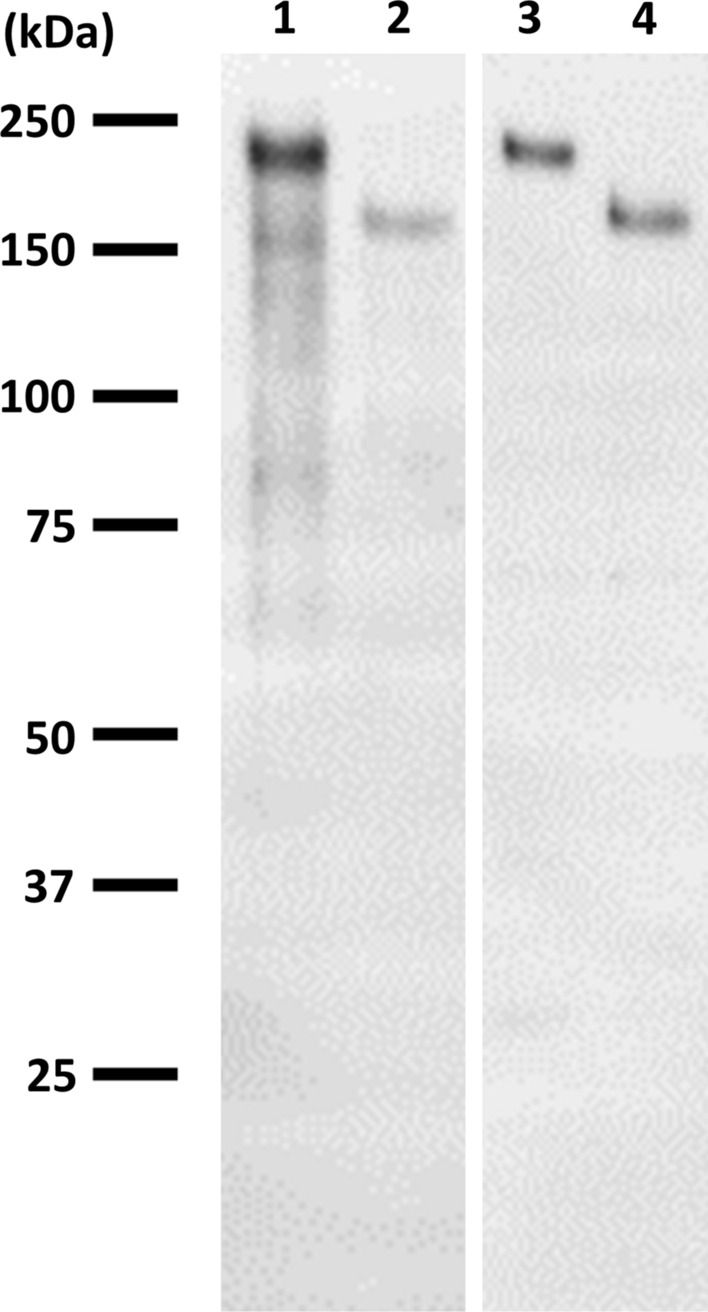
Verification of ATXN2 antibodies. Two different ATXN2 antibodies were used in this study. Their specificities were confirmed by western blotting of cultured cells transfected with ATXN2 expression plasmids, pFN21A-Halo-ATXN2 (lane 1, 3) and pcDNA3-Flag-ATXN2 (lane 2, 4). The left blot (lane 1, 2) and the right one (lane 3, 4) were incubated with mouse monoclonal antibody (BD Biosciences, aa713-904) and rabbit polyclonal antibody (Thermofisher, aa1293-1313), respectively. Halo- and Flag-tagged ATXN2 were electrophoresed at approximately 220 kDa and 160 kDa, respectively

For this study, we employed two commercial anti-ATXN2 antibodies: a monoclonal (611378; BD Biosciences) and a polyclonal (#PA5-78845; Thermofisher). We confirmed their specificity for ATXN2 using the western blotting of cultured cells transfected with different ATXN2 expression plasmids (Fig. 1). Then, we compared the immunoreactivity of these antibodies on normal human brain tissues using two different fixation and preservation methods and two antigen retrieval methods (Additional file 1: Figure S1). ATXN2-immunoreactivity was found in neuronal perikarya by all methods and in their proximal dendritic processes in all except formaldehyde-fixed, paraffin-embedded tissue. Neuronal ATXN2-immunoreactivity was highest in paraformaldehyde-fixed, free-floating sections using antigen retrieval with Tris–EDTA buffer, pH 9.0. Although comparable immunostaining was observed with both antibodies, the monoclonal antibody showed lower background staining than the polyclonal antibody. Therefore, we decided to use the monoclonal antibody and paraformaldehyde-fixed, free-floating sections using antigen retrieval with Tris–EDTA buffer, pH 9.0 for the following immunohistochemical examinations.

**Fig. 2:**
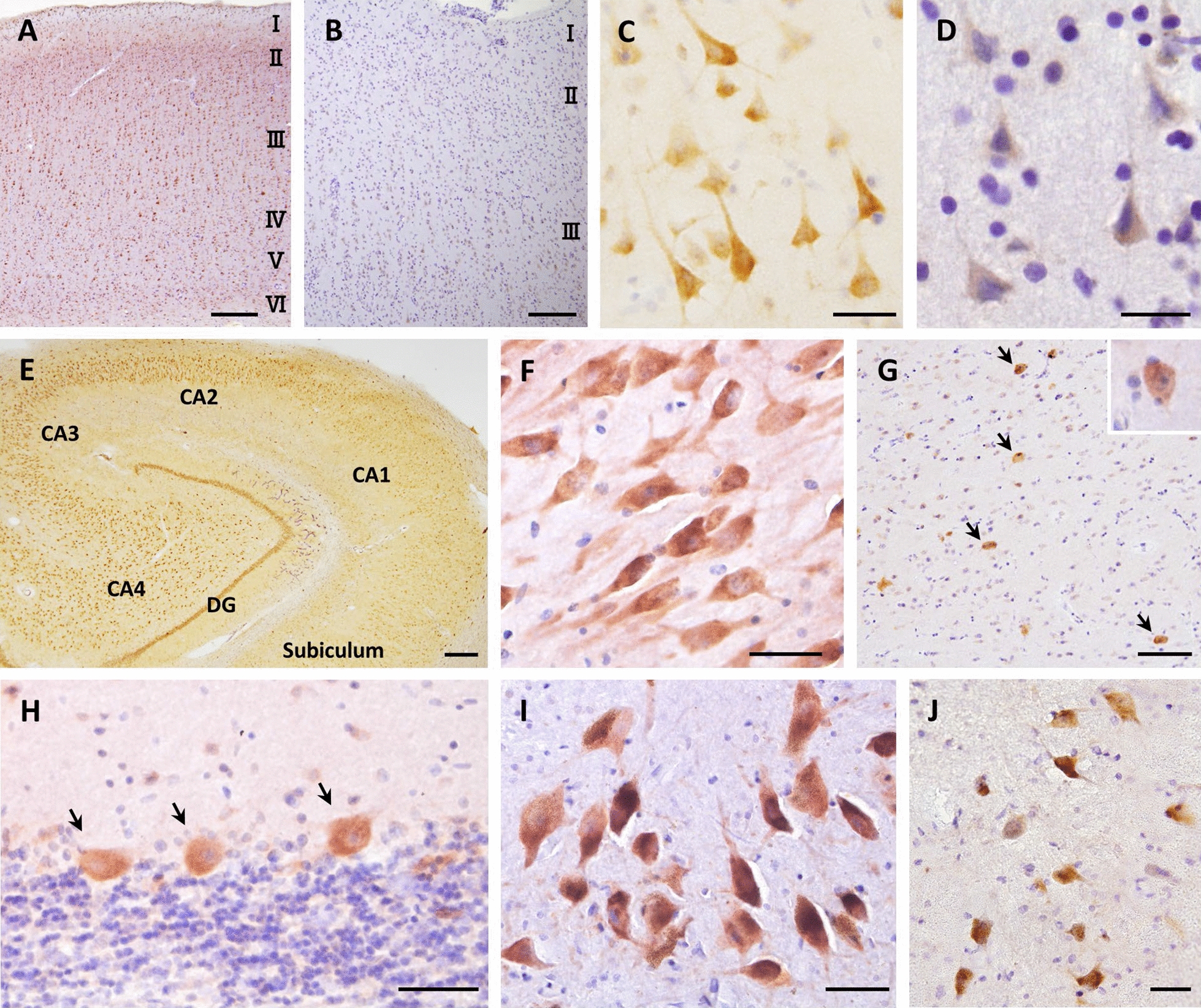
Distribution of ATXN2 in normal and FTLD-TDP human brain. Immunohistochemical labeling of normal control brain revealed the widespread distribution of ATXN2 in the neurons in the neocortex (**a**, **c**), limbic system, basal ganglia, cerebellum, midbrain, and anterior horn. **a** Low magnification image of the temporal neocortex showed neuronal ATXN2-immunoreactivity in II–VI laminae. **c** High magnification image of the III lamina. This image showed moderate neuronal ATXN2-immunoreactivity as a diffuse or granular appearance in perikarya and proximal apical and basal dendrites. The neuronal nuclei were mostly immunonegative. **e** Low magnification image of the hippocampal CA1-4 subfields and dentate gyrus, which showed the widespread distribution of neuronal ATXN2-immunoreactivity with less expression in the CA1 region. **f** High magnification image of the hippocampal CA3 showed strong ATXN2-immunoreactivity along with striated or dot appearances in the neuropil. **g** Mid magnification image of the striatum. The ATXN2-immunoreactivity in large interneurons (arrows and inset) was stronger than that in medium-size spiny neurons. **h** High magnification image of the Purkinje cells presenting strong ATXN2-immunoreactivity. **i** High magnification image of the substantia nigra pars compacta showing strong neuronal ATXN2-immunoreactivity. The neuropil was also immunolabeled as striated or dot appearances. **j** High magnification image of the anterior horn cells showed strong ATXN2-immunoreactivity. In the FTLD-TDP brain, the ATXN2-immunoreactivity was markedly decreased (**b**, **d**). **b** Low magnification image of the upper layer of the temporal neocortex showing the reduction of ATXN2-immunoreactivity in the pyramidal neurons, compared with that in controls shown in **a**. **d** High magnification image of the remaining neurons in the III lamina showed that the intracellular distribution of ATXN2 was not altered in diseased brains. The scale bars included in each image represent 50 µm (**c**, **d**, **f**, **h**, **i**, **j**), 100 µm (**g**), and 200 µm (**a**, **b**, **e**)

We then analyzed the distribution of ATXN2 positive neurons in normal human brains (Fig. 2a, c, e–j). They were observed in all areas examined, including the cerebral neocortex (Fig. 2a, c), hippocampal CA1-4 subfields and dentate gyrus (Fig. 2e, f), striatum (Fig. 2g), Purkinje cells (Fig. 2h, arrows), substantia nigra pars compacta (Fig. 2i), and anterior horn cells (Fig. 2j). ATXN2 was immunolabeled as a diffuse or granular appearance in neuronal perikarya. Their proximal apical and basal dendrites were also immunolabeled, especially in the neocortex, hippocampal CA2-4, and substantia nigra (Fig. 2c, f, i). Glial cells and axons were mostly negative. In the neocortex, ATXN2-immunoreactivity was intense in II–VI laminae where it mainly localized to pyramidal neurons, especially to the relatively large ones in the III and V laminae (Fig. 2a), while it tended to be weak in the small stellate-like neurons in the II and IV laminae. There was virtually no difference in ATXN2-immunoreactivity between the frontal and temporal cortices. In the hippocampus, ATXN2-immunoreactivity was weaker in the CA1 region than in other regions (Fig. 2e). The neuropil was also labeled by striated or dot appearances, especially in the hippocampal CA2–4 and substantia nigra pars compacta (Fig. 2e, i). In the striatum, ATXN2-immunoreactivity was more intense in large interneurons (Fig. 2g, arrows and inset) than in medium-size spiny neurons. These results indicate the widespread and mostly uniform expression of ATXN2 in normal human brains except some neuronal populations in the hippocampus and striatum.

Next, we investigated the immunoreactivity of ATXN2 in the brains of three cases with FTLD-TDP. Notably, a decreased ATXN2 immunoreactivity in the neocortical neurons was seen in all FTLD-TDP cases examined, compared to the control cases, even when both groups were simultaneously immunostained in the same operation (Fig. 2b). ATXN2 in the remaining neurons localized to neuronal perikarya and proximal dendrites (Fig. 2d), similar to that observed in the control cases.

### ATXN2 colocalizes with ribosomal protein and PABP1 in the human brains

**Fig. 3:**
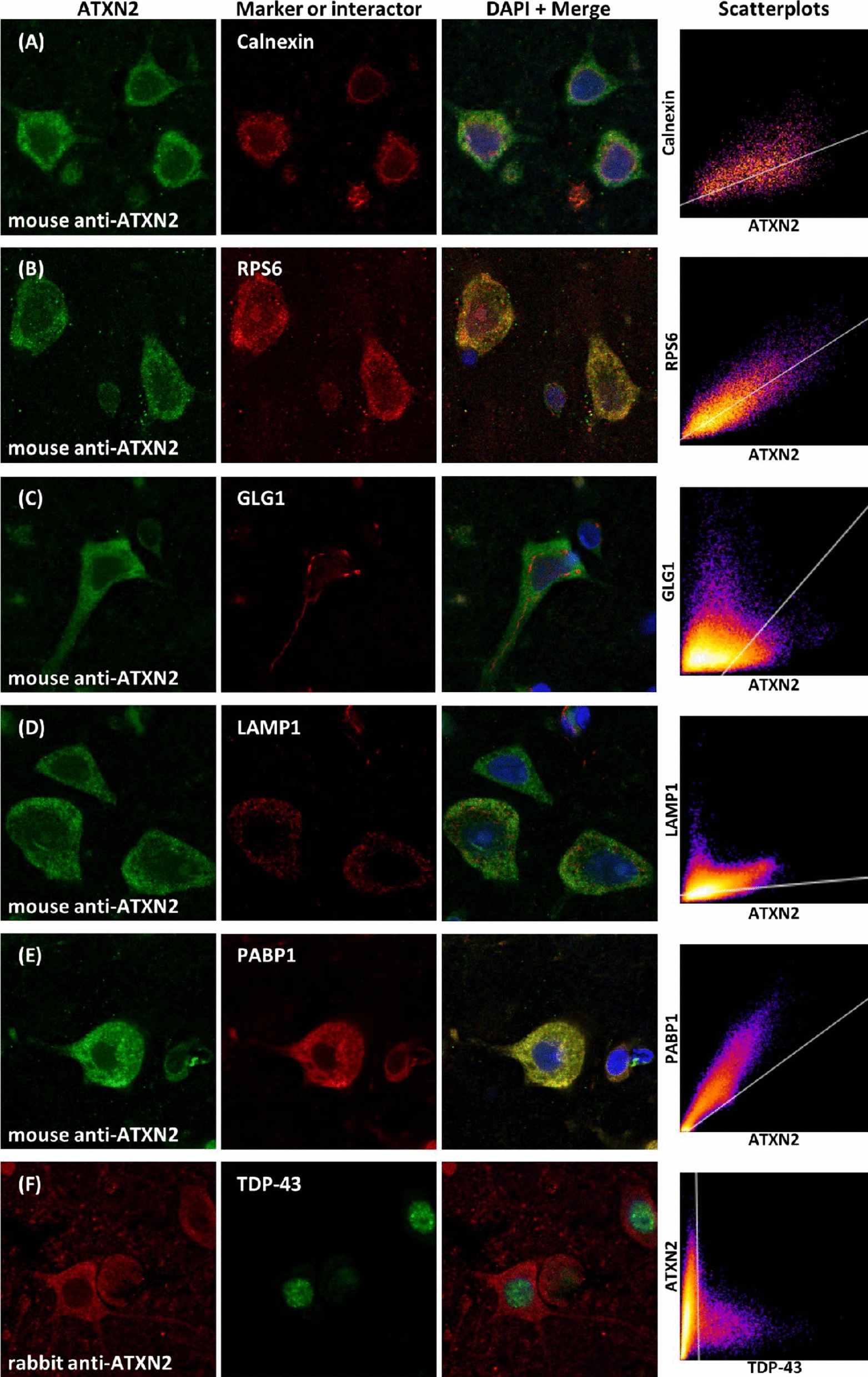
Colocalization images of ATXN2 in cytoplasmic organelle and related molecules in normal control brain. The confocal microscopic images of cytoplasmic ATXN2 and several organelle marker proteins, including endoplasmic reticulum marker Calnexin, ribosomal protein S6 (RPS6), Golgi Glycoprotein 1 (GLG1), Lysosome associated membrane protein 1 (LAMP1), ATXN2’s related protein poly-A binding protein 1 (PABP1) and TDP-43, are shown. **a**–**f** Representative fluorescent double-stained images from the upper layer of the cerebral temporal cortex, including merged images with DAPI staining and two-dimensional scatterplots. ATXN2 is partially localized within the endoplasmic reticulum (**a**) and is strongly associated with ribosomes (**b**) and PABP1 (**e**) in controls, while ATXN2 did not localize within the Golgi apparatus (**c**) and lysosomes (**d**). There was no colocalization between ATXN2 and TDP-43 (**f**). Pseudo coloring was used for each channel

To determine the subcellular organelle containing ATXN2, we conducted double-immunofluorescent analysis utilizing several antibodies to organelle markers, including the ER marker Calnexin, the ribosomal marker RPS6, the Golgi apparatus marker GLG1, and the lysosome marker LAMP1. We also analyzed the association of two proteins, PABP1 and TDP-43, which have been reported to interact with ATXN2 [4, 10, 11, 31]. In the upper layer of the temporal neocortex, hippocampal CA1, subiculum, and parahippocampal gyrus from the brains of three control cases, double-stained fluorescent images were prepared and the mean colocalization coefficients were evaluated. Representative fluorescent images from the temporal neocortex of control cases are shown in Fig. 3. Far-red channels were also observed to check for autofluorescence. The results of the colocalization analysis are graphically represented in two-dimensional scatterplots in Fig. 3 and as a summary of the PCC and tM in Table 3.

**Table 3:** Colocalization coefficient values from all evaluated brain regions of the control cases

Brain regions	Coefficients	Molecules immunolabeled with ATXN2					
		Calnexin	RPS6	LAMP1	GLG1	PABP1	TDP-43
Temporal cortex	PCC	0.4024	*0.7520*^*#*^	0.0683	0.0717	*0.7388*^*#*^	− 0.3033
	(SD)	0.0761	0.1161	0.0719	0.0544	0.0867	0.0232
	tM	0.7261*	0.9399**	0.4245	0.5203	0.897**	0.1353
	(SD)	0.0788	0.0486	0.1004	0.0501	0.0969	0.0601
Hippocampal CA1	PCC	0.4050	*0.6021*^*#*^	− 0.0423	− 0.1191	*0.7289*^*#*^	− 0.0024
	(SD)	0.1060	0.1817	0.1583	0.1720	0.0969	0.2114
	tM	0.7310*	0.8833**	0.4607	0.4284	0.9324**	0.4782
	(SD)	0.0340	0.0719	0.0277	0.1983	0.0239	0.0303
Subiculum	PCC	0.4559	*0.6872*^*#*^	− 0.0366	− 0.2399	*0.7087*^*#*^	− 0.1173
	(SD)	0.1315	0.0998	0.0265	0.1177	0.1319	0.2762
	tM	0.8064**	0.9257**	0.5100	0.3347	0.9237**	0.4434
	(SD)	0.0846	0.0178	0.0842	0.0958	0.0392	0.0625
Parahippocampal gyrus	PCC	0.4421	*0.6875*^*#*^	0.1103	− 0.1156	*0.7730*^*#*^	− 0.1112
	(SD)	0.0727	0.0360	0.1564	0.1005	0.1309	0.1103
	tM	0.7512*	0.9218**	0.6145	0.4643	0.9627**	0.4236
	(SD)	0.1079	0.0243	0.0624	0.0704	0.0219	0.0355

Pearson’s correlation coefficients above threshold (PCC) and Manders’ colocalization coefficients above threshold (tM) of control cases were calculated according to each cytoplasmic ROI. Each mean value from at least fifteen ROIs was plotted with standard deviation. PCC represents the overall linear relationship from − 1 (perfectly inversely related) to 1 (perfectly related). Any value greater than − 0.5 and less than 0.5 is taken to mean that the two probes are unrelated. PCC values showing a significant relation are indicated in italics letters with ^#^ in the chart. tM shows the fraction of ATXN2’s fluorescence in compartments containing fluorescence of its counterpart molecule and ranges from 0 to 1. We classified a tM as high (0.8 ≤ tM, indicated in italics letters with ** in the chart), moderate (0.65 ≤ tM < 0.8, indicated in italics letters with *), or low (tM < 0.65, no highlights)

ATXN2 partially colocalized with Calnexin, an ER marker, and its overlap was observed in some parts of the peripheral ER but not in nuclear envelops stained with anti-Calnexin (Fig. 3a). Reflecting the partial overlap of these proteins, the colocalization analysis did not reach a significant linear relation (0.40 < PCC < 0.46), while the tM rates, which indicate the overlap rate of ATXN2-immunoreactivity with Calnexin-immunoreactivity, showed a moderate value (around 0.75). These results indicate that ATXN2 is present in the peripheral ER but is more widely distributed in other subcellular areas. In contrast, ATXN2 overlapped with ribosomal protein RPS6 in most parts of the cytoplasm (Fig. 3b). Indeed, their coefficients showed a significant linear relation (0.60 < PCC < 0.75) and the tM rates of ATXN2 to RPS6 was above 90%, suggesting a strong association between both proteins. ATXN2 had almost no overlap with GLG1 and LAMP1, which are Golgi apparatus and lysosomal markers, respectively (Fig. 3c, d), and their PCC and tM rates did not show any relevant values (− 0.5 < PCC < 0.5 and tM < 0.65). PABP1-immunoreactivity showed a cytoplasmic localization similar to that of ATXN2, and overlaps of ATXN2 and PABP1 were observed throughout the cytoplasm (Fig. 3e). ATXN2 showed a high correlation with PABP1 (0.70 < PCC < 0.78), similar to that with RPS6, and the tM of both proteins was over 90%. In contrast, there was no colocalization in the double-fluorescence labeling using rabbit polyclonal anti-ATXN2 antibody and mouse monoclonal anti-TDP-43 antibody. The immunofluorescent signal of each antibody was mainly localized in the cytoplasm and nucleus, respectively (Fig. 3f), and no correlation was obtained in the analysis.

**Fig. 4:**
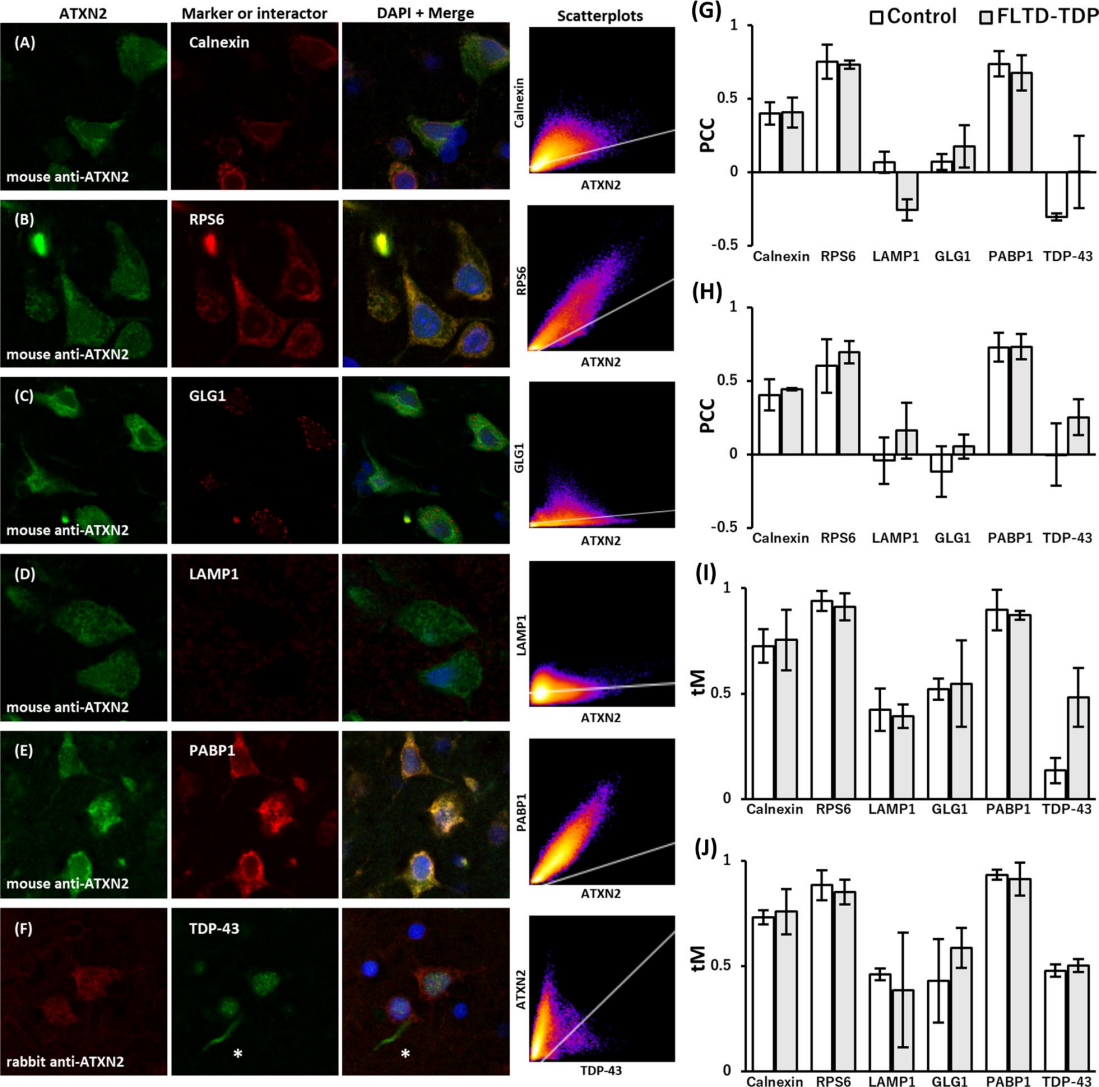
Comparison of ATXN2 localization in the neuronal cytoplasm in control and FTLD-TDP brains. **a**–**f** The confocal microscopic images of neuronal cytoplasmic ATXN2 and organelle marker proteins or ATXN2’s related proteins from a FTLD-TDP patient’s brain were obtained as were those of controls. Representative fluorescent double-stained images from the upper layer of the cerebral temporal cortex, including merged images with DAPI staining and two-dimensional scatterplots, are presented. The localization of ATXN2 in the neuronal cytoplasm did not differ from that in the controls shown in Fig. 3. Note that the LAMP1-immunoreactivity was strikingly decreased (**d**). **f** In the double-fluorescent images using the rabbit polyclonal anti-ATXN2 antibody and the mouse monoclonal phosphorylation-independent anti-TDP-43 antibody, asterisks indicate that ATXN2 did not appear in TDP-43 positive dystrophic neurites. Pseudo coloring was used for each channel. **g**–**j** Colocalization coefficient values for the upper layer of the temporal cortex (**g**, **i**) and hippocampal CA1 (**h**, **j**) were obtained from normal controls and FTLD-TDP patients. The mean values of Pearson’s correlation coefficients above threshold (PCC) (**g**, **h**) and Manders’ colocalization coefficients above threshold (tM) (**i**, **j**) are plotted. ATXN2 and Calnexin did not show significant PCC rates but moderate tM rates, indicating their fractional overlapping. RPS6 and PABP1 showed both significant PCC rates and high tM rates with ATXN2, indicating their linear relation and strong overlapping. LAMP1, GLG1, and TDP-43 showed the most insignificant PCC rates and the lowest tM rates with ATXN2. Those coefficients were not very different between the controls and FTLD-TDP cases

We next similarly analyzed the double-stained fluorescent images and colocalization coefficients in the brain of three FTLD-TDP cases and compared the results with those in the normal controls. While ATXN2-immunoreactivity tended to be weaker in the FTLD-TDP cases than in the controls, the localization pattern of ATXN2 in the neuronal cytoplasm did not differ from that in the controls (Fig. 4a–f), which was also confirmed by no large differences in the PCC and tM coefficient values between the two groups as shown in Fig. 4g–j. The coefficient values of FTLD-TDP cases are summarized in Table 4. Of the marker proteins examined in this study, LAMP1-immunoreactivity was strikingly decreased in all evaluated regions in FTLD-TDP cases, consistent with a previous report [2], while the colocalization rates of LAMP1 with ATXN2 were not altered (− 0.26 < PCC < 0.17 and tM < 0.50).

**Table 4:** Summary of colocalization coefficient values from all evaluated brain regions of the FTLD-TDP cases

Brain regions	Coefficients	Molecules immunolabeled with ATXN2					
		Calnexin	RPS6	LAMP1	GLG1	PABP1	TDP-43
Temporal cortex	PCC	0.4075	*0.7336*^*#*^	− 0.2568	0.1767	*0.6783*^*#*^	0.0027
	(SD)	0.1026	0.0293	0.0715	0.1448	0.1207	0.2457
	tM	0.7545*	0.9126**	0.3935	0.5475	0.872**	0.4817
	(SD)	0.1427	0.0640	0.0560	0.2057	0.0209	0.1394
Hippocampal CA1	PCC	0.4421	*0.6949*^*#*^	0.1620	0.0542	*0.7327*^*#*^	0.2516
	(SD)	0.0080	0.0752	0.1907	0.0824	0.0854	0.1221
	tM	0.7582*	0.8505**	0.3861	0.5867	0.9128**	0.5021
	(SD)	0.1067	0.0591	0.2732	0.0952	0.0793	0.0309
Subiculum	PCC	*0.582*^*#*^	*0.6819*^*#*^	− 0.0167	− 0.0019	*0.7626*^*#*^	0.1219
	(SD)	0.0942	0.0556	0.0811	0.0989	0.1431	0.0774
	tM	0.6010	0.7563*	0.4915	0.6462	0.9436**	0.4522
	(SD)	0.1824	0.1796	0.1059	0.1177	0.0330	0.1347
Parahippocampal gyrus	PCC	0.4842	*0.7329*^*#*^	− 0.0699	0.1045	*0.8244*^*#*^	0.1056
	(SD)	0.0405	0.0413	0.0495	0.1657	0.0281	0.1676
	tM	0.7506*	0.8092**	0.4537	0.5529	0.9393**	0.3906
	(SD)	0.0738	0.1445	0.1079	0.0878	0.0410	0.1287

The mean values of Pearson’s correlation coefficients above threshold (PCC) and Manders’ colocalization coefficients above threshold (tM) of FTLD-TDP cases were calculated and plotted by the same method as for the normal controls and presented no large differences from the results in controls (Table 3). For the meaning of the symbols in the table, refer to the explanation in Table 3

### Colocalization of ATXN2 and phosphorylated TDP-43 in DNs and NCIs in FTLD-TDP cases

**Fig. 5:**
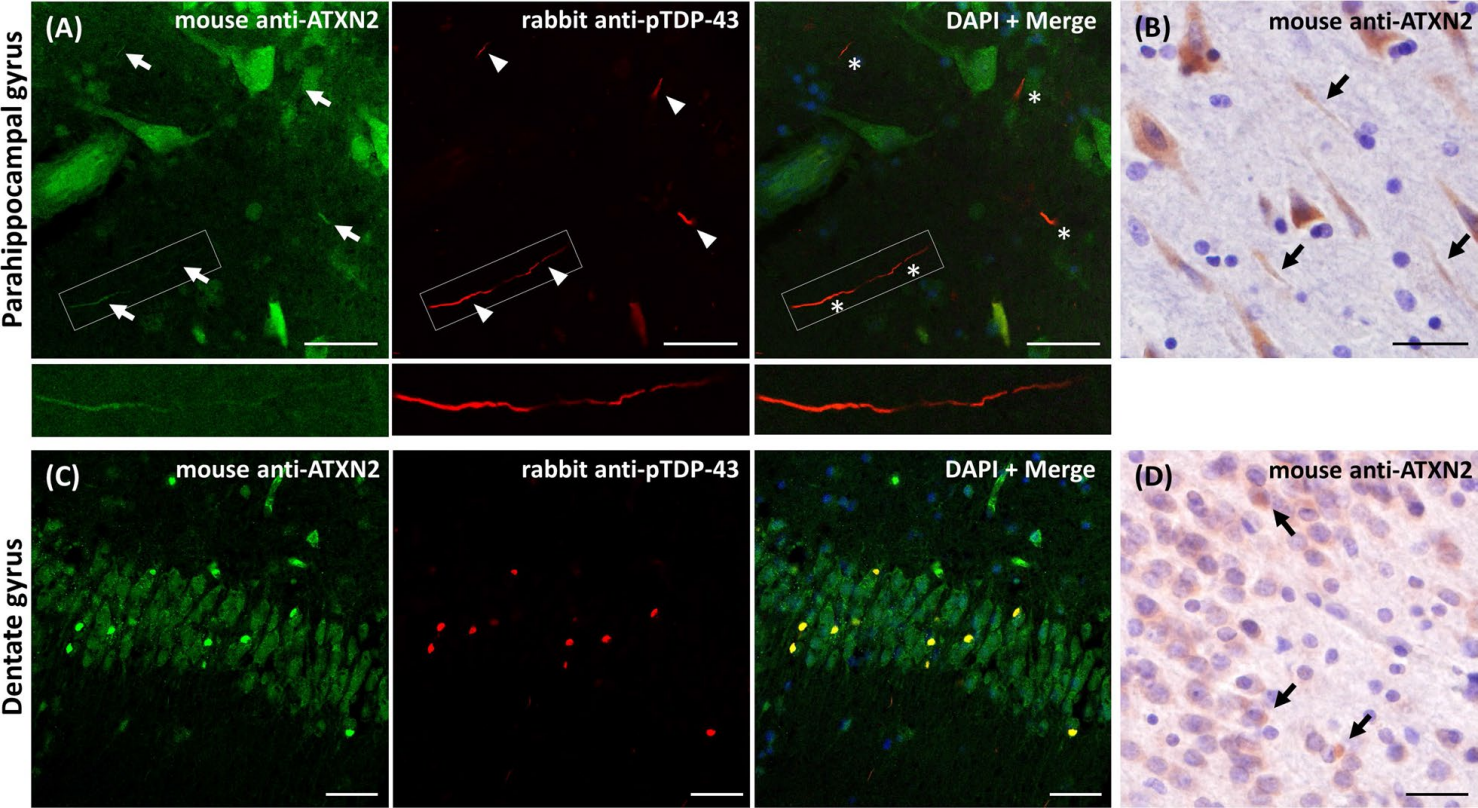
Colocalization image of ATXN2 and pTDP-43 in the dentate gyrus of FTLD-TDP brain. The parahippocampal gyrus and dentate gyrus of FTLD-TDP cases were immunostained by double-immunofluorescent labeling using the mouse monoclonal anti-ATXN2 antibody and the rabbit polyclonal anti-pTDP-43 antibody or immunoperoxidase labeling using the mouse monoclonal anti-ATXN2 antibody. Each representative image is presented. **a** The double-immunofluorescent images of parahippocampal gyrus showed that part of pTDP-43 positive DNs (red image, arrowheads) were positive to ATXN2 (green image, arrows), and that both signals colocalized in the merged image (asterisks). Representative DN is also presented in the magnified images. **b** The immunoperoxidase labeling of parahippocampal gyrus showed a weak ATXN2-immunoreactivity resembling TDP-43 positive DNs (arrows). **c** The double-immunofluorescent images of the dentate gyrus showed the colocalization of pTDP-43 and ATXN2 in most of NCIs formed in the granule cells. **d** The immunoperoxidase labeling of the dentate gyrus showed a weak ATXN2-immunoreactivity resembling TDP-43 positive NCIs (arrows). In **a** and **c**, DAPI staining is included in the merged image, and pseudo coloring was used for each channel. The scale bars included in each image represent 30 µm

Double-immunofluorescent staining using mouse monoclonal anti-ATXN2 antibody and rabbit polyclonal anti-pTDP-43 antibody showed that part of pTDP-43 positive DNs were positive for ATXN2 in the parahippocampal cortex of FTLD-TDP cases (Fig. 5a, arrows and magnified images). After this finding, we re-observed the sections of the same region stained with DAB and could found the ATXN2 positive structures resembling DNs (Fig. 5b). Similarly, double-immunofluorescent staining using the same antibody combination revealed that most of pTDP-43 positive NCIs in the granule cells of the dentate gyrus was positive for ATXN2 (Fig. 5c), which allowed us to recognize ATXN2 positive NCIs in the sections of the same region stained with DAB (Fig. 5d).

### ATXN2 associates with coupled ribosomes in mammalian brain

**Fig. 6:**
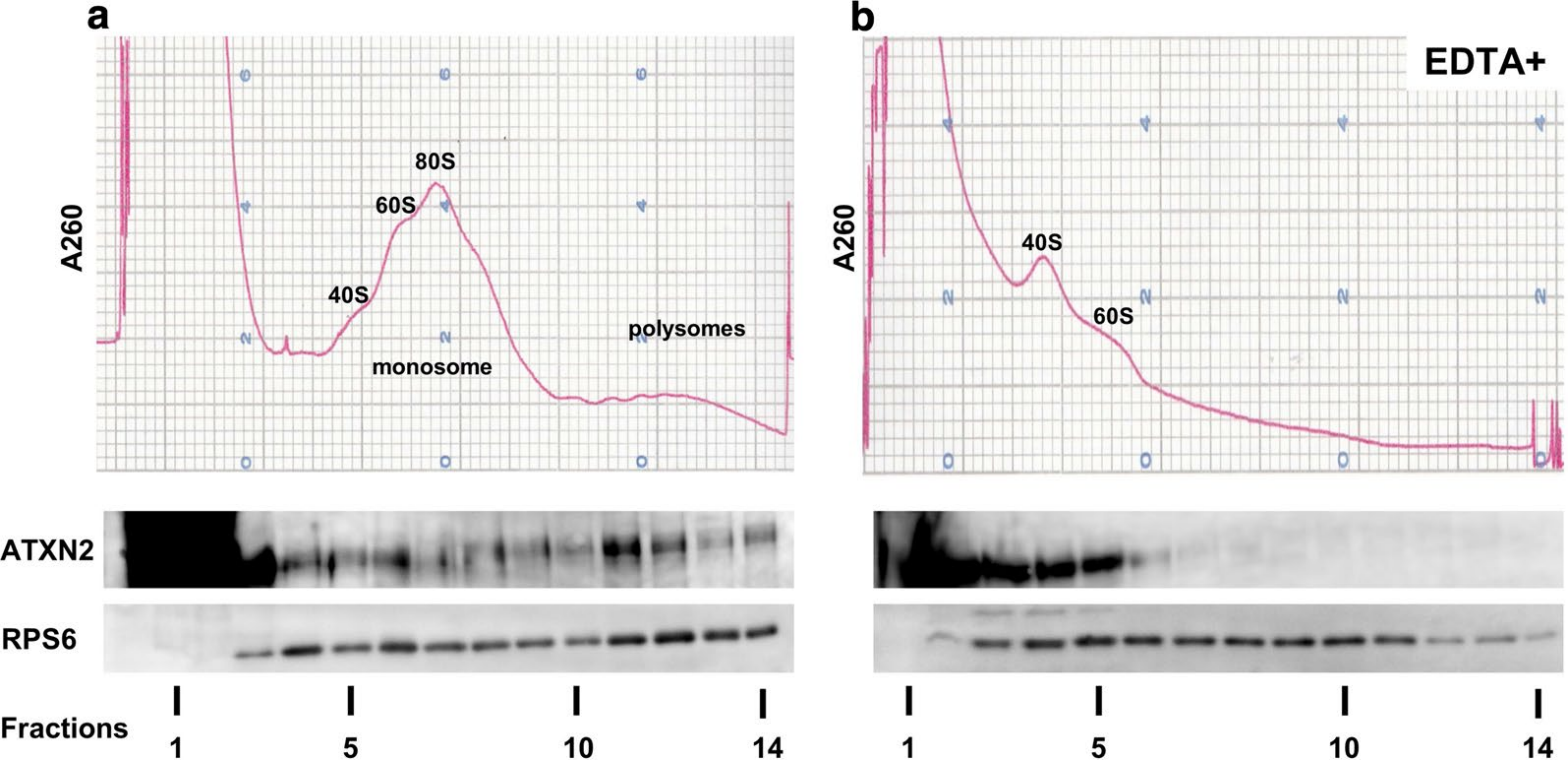
Polysome profiling of ATXN2 of mouse brain homogenates. Mouse whole brain homogenates were fractionated in a 10–50% (w/v) sucrose density-gradient, collected with a monitoring RNA absorption curve (absorbance at 260 nm: A260), and analyzed by western blotting. **a** Analysis of whole brain extract from a mouse showed the sedimentation of ATXN2 and ribosomal subunit RPS6 in both monosomal and polysomal fractions. **b** EDTA treatment disrupted the sedimentation of ATXN2 and RPS6 with polysomal fractions. The cropped blots are presented for clarity and conciseness

In order to investigate the biochemical associations between ATXN2 and ribosomes, we examined polysome (coupled-ribosome) profiles on linear sucrose density-gradients, in which we used samples extracted from SH-SY5Y cells (Additional file 2: Figure S2) or mouse brains (Fig. 6) to avoid an effect of RNA degradation due to typical post-mortem delay in human tissue samples. The RNA absorbance curve and western blotting analysis of each fraction is shown in Fig. 6a and Additional file 2: Figure S2a. In both extracted lysates from cultured cells and mouse brains, we found ATXN2 sediments in both monosomal and polysomal fractions, overlapping with RPS6. To confirm this result, the same samples were lysed in a polysome lysis buffer containing EDTA in place of MgCl_2_. EDTA chelates Mg^2+^ ions and dissociates the large and small ribosomal subunits [9]. Our EDTA treatment disrupted polysomes and 80S monosomes, and they converted to a lighter fraction of gradient sucrose as presented on the RNA absorbance curve. Also, the marked decrease of ATXN2 and RPS6 bands in polysomal fractions was detected by western blotting (Fig. 6b and Additional file 2: Figure S2b). The shifting in absorption profile and sedimented protein corresponded with similar previous studies of ATXN2 or another RBP which also associates with ribosomes [37, 39]. Our results confirmed that endogenous ATXN2 associates with mammalian translational machinery through binding to polysomes in vitro and in vivo.

### The level of ATXN2 is decreased in the FTLD-TDP human brain neurons

**Fig. 7:**
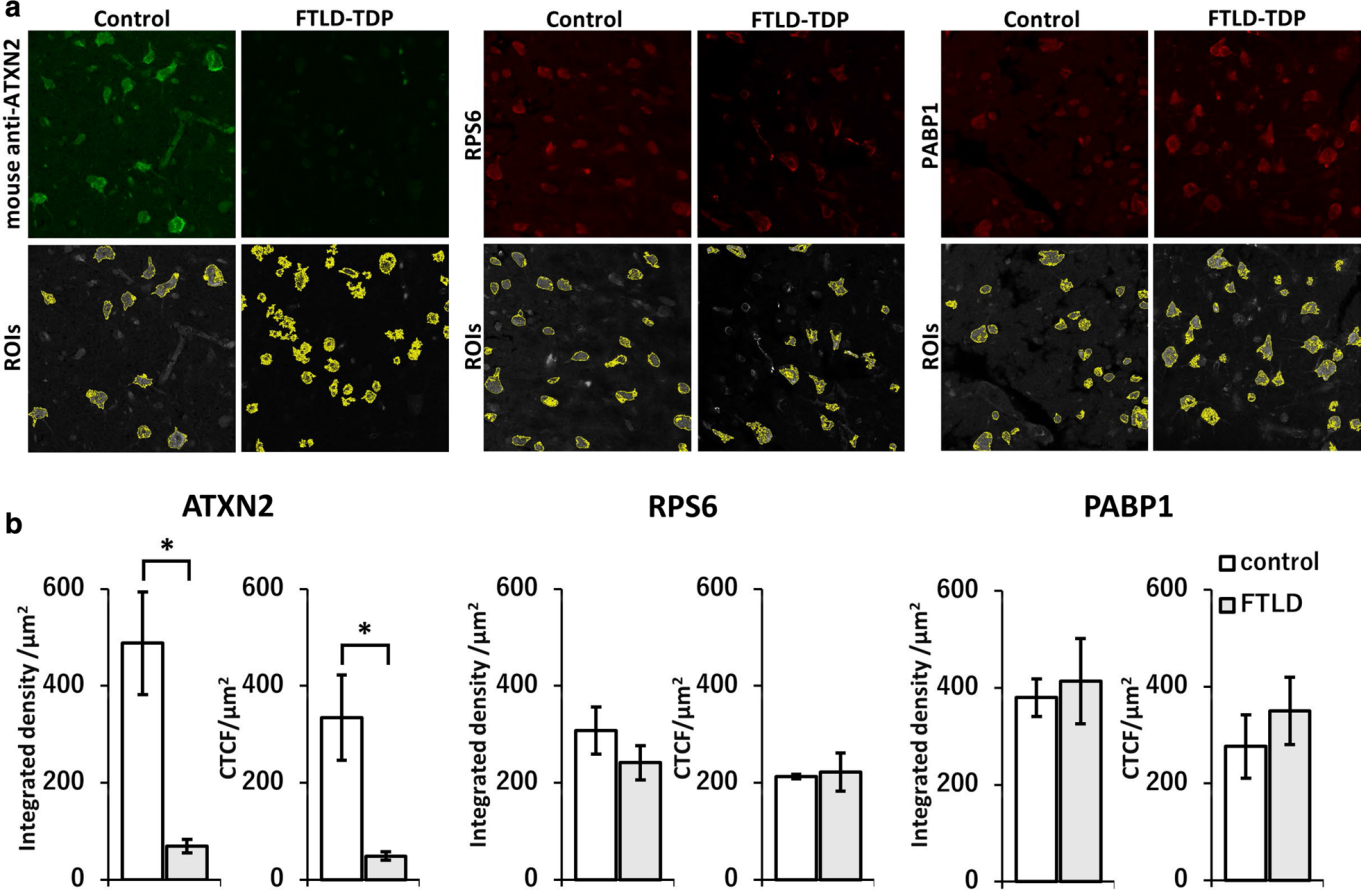
Semi-quantitative immunohistochemical analysis of human neuronal ATXN2. The brain sections of temporal neocortex from three control cases and three FTLD-TDP cases were immunostained with the antibodies for ATXN2, RPS6, and PABP1 by the same procedure. The signal intensities within each neuronal cytoplasm from the fluorescent images were semi-quantified and statistically compared. **a** Representative immunofluorescent images and automatically determined ROIs within each neuronal cytoplasm. **b** Integrated density and corrected total cell fluorescence (CTCF) values per unit area (μm^2^) for ATXN2, RPS6, and PABP1 were calculated from fifty ROIs and plotted. Data are mean ± standard deviation, and statistical comparisons were made by unpaired Student T tests. The signal intensity of ATXN2 in the FTLD-TDP cases was significantly reduced compared with controls (*P ≤ 0.05), but its related molecules RPS6 and PABP1, showed no such significant reduction

As shown in Fig. 2, we noticed a weaker immunohistochemical signal of ATXN2 in the FTLD-TDP cases compared with normal controls. It was speculated that the expression level of endogenous ATXN2 decreases in association with its involvement in TDP-43 pathophysiology. To confirm this, we compared the semi-quantification of the fluorescent intensity of ATXN2 and its related proteins, RPS6 and PABP1, in the temporal neocortex between three controls and three FTLD-TDP cases, which were immunostained together. Quantification of the integrated density and CTCF within neuronal cytoplasmic ROIs revealed that the signal intensity of ATXN2 was significantly lower in the FTLD-TDP cases compared with controls, while there were no significant differences in the signal intensity of RPS6 and PABP1 between the two (Fig. 7).

**Fig. 8:**
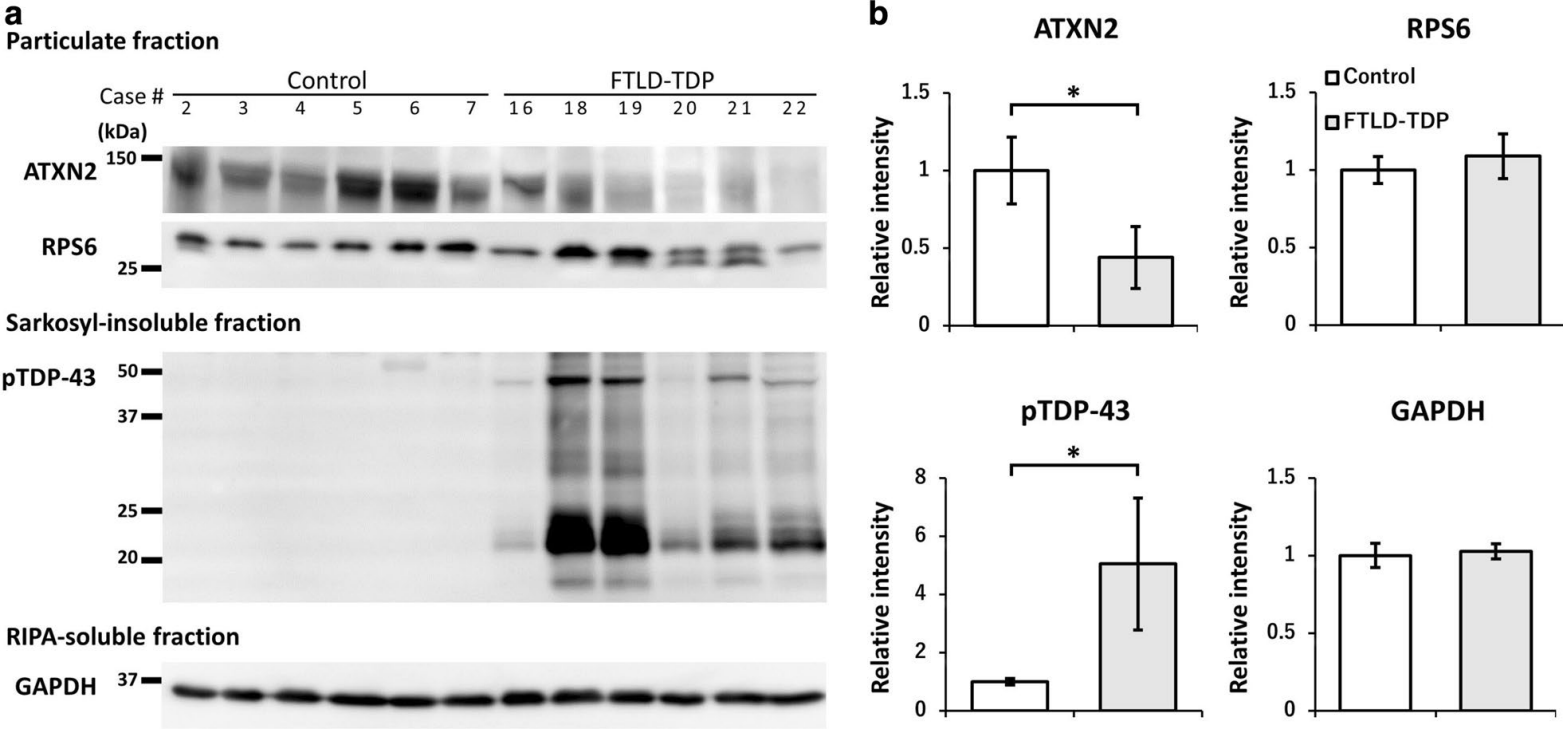
Semi-quantitative western blotting analysis of human brain ATXN2. Brain homogenates from six normal controls and six FTLD-TDP cases were analyzed by western blotting, and each band intensity was quantified and statistically compared. **a** Immunoblots of each sample detected by the antibodies for ATXN2, RPS6, pTDP-43, and GAPDH. The blots were cropped for clarity and conciseness. **b** Semi-quantifications of band levels in each group were calculated and plotted. Data are mean ± standard deviation, and statistical comparisons were made by the Mann–Whitney U tests. The level of ATXN2 in FTLD-TDP cases was significantly decreased compared with controls (*P < 0.01). On the other hand, the levels of RPS6 and GAPDH were not largely changed between two groups. The bands of pTDP-43 in sarkosyl-insoluble fraction were found only in FTLD-TDP cases

Furthermore, we compared the expression level of ATXN2 by biochemical analyses using brain homogenates from six controls and six FTLD-TDP cases (Fig. 8). The representative immunoblot analyses of each case using antibodies for ATXN2, RPS6, pTDP-43, and GAPDH are shown in Fig. 8a. The semi-quantification of these bands and comparison of each group are shown in Fig. 8b. The level of ATXN2 was significantly decreased in the FTLD-TDP cases compared with the controls. There was no strong correlation between the quantified ATXN2 bands and the pTDP-43 bands by the immunoblot of the six FTLD-TDP brains (PCC = 0.25). For comparison, the band levels of RPS6 were similarly analyzed and showed no significant difference between the two groups. The bands of pTDP-43 in sarkosyl-insoluble fraction were found only in FTLD-TDP cases. GAPDH was also analyzed as a loading control. The immunoblot of ATXN2 in Fig. 8a is also entirely presented in Additional file 3: Figure S3A. ATXN2 was not present in sarkosyl-insoluble fractions of both normal controls and FTLD-TDP cases (Additional file 3: Figure S3B).

## Discussion

To clarify the role of ATXN2 in the pathogenesis of FTLD-TDP, we examined the intracellular dynamics of ATXN2 in brains of normal controls and FTLD-TDP cases. Our sensitive immunohistochemical methods made it possible to reveal the detailed anatomical distribution and subcellular localization of ATXN2 in human brains for the first time, since previous studies were mostly limited to in vitro experiments or animal studies [4, 10, 19, 31, 35, 37, 42], and the immunohistochemical studies of ATXN2 in normal human brains employed formalin-fixed and paraffin-embedded sections with low sensitivity [11, 17].

First, the present study clarified the regional and subcellular localization of ATXN2 in normal human brains. Huynh et al. showed the widespread distribution of neuronal ATXN2 immunoreactivity in normal human brains, including the frontal cortex, hippocampus, basal ganglia, midbrain, medulla, and cerebellum, with a higher immunoreactivity in Prukinje cells than in other cell types [17]. The wide distribution of neuronal ATXN2 immunoreactivity observed in this study is consistent with their results except that the staining intensity of Prukinje cells was virtually comparable to that of most other neuronal cells. No difference in ATXN2 immunoreactivity between the frontal and temporal cortices in this study is also consistent with the previous report that showed an equivalent level of human ATXN2 gene expression in both regions [20]. One of the new findings in this study is that the intensity of ATXN2 immunostaining differs in some neuronal populations in the hippocampus and striatum. Moreover, extended ATXN2 immunoreactivity to the proximal apical and basal dendrites observed in many regions in our study differs from that confined to the neuronal cytoplasm in the study by Huynh et al. [17]. Such peripheral subcellular localization of ATXN2 found in this study may be consistent with the recent report that indicates roles for ATXN2 in the assembly of RNP granules and their cellular transport [3]. Thus, our findings may indicate multiple functions of ATXN2 in many subcellular regions.

The detailed subcellular localization of ATXN2 in normal human brains was further clarified by double immunofluorescence staining in this study. Colocalization analysis revealed that RPS6, a ribosomal marker, and PABP1, a putative factor promoting ATXN2 association with polyribosome [4, 10, 31, 35, 37], were strongly associated with ATXN2. These results suggest the localization of ATXN2 in ribosomes. The evidence of direct binding of ATXN2 to polysomes, which consist of two or more ribosomes, shown by polysome profiles analysis using cultured cells and mouse brains in this study further support this notion. These results are consistent with the previous reports that showed the involvement of ATXN2 with translational machinery through ribosomes in vitro [4, 12, 37]. Furthermore, since PABP1 binds the poly-A tail of mRNA and regulates processes of mRNA metabolism such as pre-mRNA splicing and mRNA stability [35], ATXN2 may be involved in such processes in combination with PABP1 in the human brain. Additionally, because of several previous studies using cultured cells, mice, and human brain homogenates that showed localization of ATXN2 in the ER and Golgi apparatus, we examined the colocalization between ATXN2 and these organelle markers and found that ATXN2 partially colocalized with the ER but not with the Golgi apparatus. Taken together, these results suggest that ATXN2 is widely distributed to the subcellular regions where ribosomes exist. This indicates that ATXN2 associates not only with membrane-bound ribosomes that produce proteins to be secreted, but also with free ribosomes producing proteins used inside the cells.

Then, we focused on whether there were ATXN2 abnormalities in the brains of patients with FTLD-TDP by immunohistochemical and biochemical experiments. The results showed that ATXN2 is not only localized to translational machinery, but also colocalized to pTDP-43 positive NCIs and DNs in brains of FTLD-TDP cases. Colocalization of ATXN2 with TDP-43 positive NCIs was previously reporetd [11], but for the first time, we found that ATXN2 is also colocalized with pTDP-43 positive DNs in FTLD-TDP brains. This observation indicates that ATXN2 is involved in the formation of TDP-43 aggregates at least in part, possibly causing acceleration of neurodegeneration as well. Consistent with this notion, genetic alteration of ATXN2 as intermediate CAG repeat expansions was reported to exacerbate the disease onset and clinical phenotype of FTLD [36]. These findings suggest that ATXN2 is involved in pathogenesis of the disease through toxic gain of function mechanisms.

We also showed a decrease in ATXN2 in the neocortex of FTLD-TDP brains. The decrease of ATXN2 may be caused by transcriptional downregulation of ATXN2 mRNA or some post translational event such as aggregation of ATXN2. Given the presence of ATXN2-immunoreactivity in some TDP-43 positive aggregates, that are major pathological structures of FTLD-TDP, the latter hypothesis is possible. However, the biochemical analysis showed that ATXN2 was not detected in the sarkosyl-insoluble fraction, suggesting that intracellular accumulation of ATXN2 would have only a limited effect on its reduction. Also, we were not able to confirm whether cytoplasmic ATXN2 staining was selectively reduced in granule cells in the presence of NCIs, because their very small cell body was unsuitable for analysis of cytoplasmic immunoreactivity. In order to address the effect of ATXN2 aggregation on its reduction, future studies must compare the ATXN2-immunoreactivity of neocortical neurons with and without NCIs using FTLD-TDP type A and type B specimens. Reverse transcription-PCR or in situ hybridization methods may be useful for analyzing ATXN2 mRNA in FTLD-TDP cases to elucidate the former hypothesis. Furthermore, to clarify the association between ATXN2 reduction and TDP-43 positive structures formation in the pathogenesis of FTLD-TDP, the expression level of ATXN2 across wide brain regions especially without TDP-43 pathology such as the occipital region and the cerebellum might be analyzed in the future.

In FTLD-ALS, sequestration from normal cellular components or deficiency of several RBPs that are produced from disease causative genes is considered to cause the pathogenic loss-of-function in RNA metabolism [8, 14]. Similarly, it may be that decreased expression of ATXN2 reduces the expression of proteins essential for neuronal function and modifies the pathological processes. Indeed, the previous study using ATXN2 knockout mice showed that genetically ablated ATXN2 enhances translation machinery via phosphorylation of RPS6 but decreases the overall protein synthesis rate [12]. However, it is unclear whether the decreased expression of ATXN2 observed in this study is enough to have a neurotoxic effect. In contrast, considering the localization of ATXN2 in the pTDP-43 positive aggregates found in this study, it is possible that ATXN2 is involved in the toxicity of TDP-43 aggregates. A recent study has shown that reduced exprssion of ATXN2 attenuates the toxicity of TDP-43 and becomes a candidate for therapeutic targets in TDP-43 proteinopathies [5]. In that study, the authors crossed ATXN2 knockout mice with TDP-43 transgenic mice and showed the decrease in ATXN2 reduced aggregation of TDP-43, markedly increased survival, and improved motor function. Moreover, administering antisense oligonucleotides targeting ATXN2 markedly extended the life span of TDP-43 transgenic mice. Therefore, there may be a possibility that ATXN2 in the brains of FTLD-TDP cases was secondarily down-regulated as to a neuroprotective effect to attenuate the neurotoxicity of TDP-43 aggregates. Finally, it would be interesting to see if colocalization of ATXN2 and pathogenic proteins and the decrease in ATXN2 expression are specific to FTLD-TDP or if they occur in other TDP-43 proteinopathies including limbic-predominant age-related TDP-43 encephalopathy and other proteinopathies such as tauopathies and alpha-synucleinopathies to elucidate the pathogenesis of these neurodegenerative disorders.

## Conclusions

The present study clarified ATXN2’s putative physiological interaction in translation, RNA stabilization, and pathological involvement in FTLD-TDP. Although it is an issue for further study as to whether the reduction of ATXN2 is neuroprotective or neurotoxic in the pathological process of FTLD-TDP, the results of the present study provide important information that should influence the strategy for new therapeutic development. Further investigations employing brains from a larger number of cases are needed to confirm our observations and to clarify the pathological role of ATXN2 in FTLD-TDP and other TDP-43 proteinopathies.

## Supplementary information

Additional file 1: Figure S1. Examination for the appropriate immunohistochemical method to detect intracellular ATXN2. The appropriate fixation and antigen retrieval methods were determined by immunoperoxidase labeling of brain sections. The highest sensitivity of neuronal anti-ATXN2 immunoreactivity was obtained using paraformaldehyde-fixed, free-floating brain sections of the cerebral temporal cortex. Also, the heating preparation with Tris–EDTA buffer, pH 9.0 worked better than that with citrate buffer, pH 6.0.

Additional file 2: Figure S2. Polysome profiling of ATXN2 in SH-SY5Y lysates. The cell lysates were fractionated in 10–50% (w/v) sucrose density-gradient, collected with a monitoring RNA absorption curve at 260 nm (A260), and analyzed by western blotting. **a** The analysis of cell lysate without EDTA showed sedimentation of ATXN2 and ribosomal subunit RPS6 in both monosomal and polysomal fractions. **b** EDTA treatment disrupted the sedimentation of ATXN2 and RPS6 in polysomal fractions. The cropped blots were presented for clarity and conciseness.

Additional file 3: Figure S3. Western blotting analysis of human brain ATXN2. Particulate fractions and sarkosyl-insoluble fractions of brain homogenates from six normal controls and six FTLD-TDP cases were analyzed by western blotting using the mouse monoclonal anti-ATXN2 antibody. Halo-tagged ATXN2 sample was also analyzed as a positive control in each blot (arrowhead). **a** Immunoblot of particulate fraction. ATXN2 was electrophoresed at approximately 150 kDa (arrow). **b** Immunoblot of sarkosyl-insoluble fraction. Insoluble ATXN2 was not found in both normal controls and FTLD-TDP cases.

## Data Availability

The datasets supporting the conclusions of this article are included within the article and its additional files.

## Abbreviations

ALS: amyotrophic lateral sclerosis
ATXN2: Ataxin-2
A260: RNA absorption curve at 260 nm
BG: basal ganglia
CTCF: corrected total cell fluorescence
DMEM: Dulbecco’s modified Eagle’s medium
DN: dystrophic neurite
ER: endoplasmic reticulum
F: frontal cortex
FA: formaldehyde
FTLD: frontotemporal lobar degeneration
FUS: fused-in-sarcoma
GAPDH: glyceraldehyde-3-phosphate dehydrogenase
GLG1: Golgi Glycoprotein 1
Hip: hippocampal region
hnRNPs: heterogenous nuclear ribonucleoproteins
IF: immunofluorescence
IPL: immunoperoxidase labeling
LAMP1: lysosome associated membrane protein 1
MND: motor neuron disease
NCI: neuronal cytoplasmic inclusion
NII: neuronal intranuclear inclusion
PABP1: poly-A binding protein 1
PBS: phosphate-buffered saline
PCC: Pearson’s correlation coefficient above threshold
PFA: paraformaldehyde
polyQ: polyglutamine
pTDP-43: phosphorylated TDP-43
RBPs: RNA-binding proteins
ROIs: regions-of-interest
RPS6: ribosomal protein S6
SC: spinal cord
SCA2: spinocerebellar ataxia type 2
T: temporal cortex
TDP-43: TAR DNA-binding protein of 43 kDa
tM: Manders’ colocalization coefficient above threshold
WB: western blotting
