# Supplementary material for: Intracellular dynamics of Ataxin-2 in the human brains with normal and frontotemporal lobar degeneration with TDP-43 inclusions
Source: Acta Neuropathol Commun. 2020 Oct 28;8:176. doi: 10.1186/s40478-020-01055-9 (PMC7594343; doi:10.1186/s40478-020-01055-9)
Supplement: Supplementary file 1 — Examination for the appropriate immunohistochemical method to detect intracellular ATXN2. The appropriate fixation and antigen retrieval methods were determined by immunoperoxidase labeling of brain sections. The highest sensitivity of neuronal anti-ATXN2 immunoreactivity was obtained using paraformaldehyde-fixed, free-floating brain sections of the cerebral temporal cortex. Also, the heating preparation with Tris–EDTA buffer, pH 9.0 worked better than that with citrate buffer, pH 6.0. [file 40478_2020_1055_MOESM1_ESM.pdf]

Additional file 1: Supplementary Fig. 1

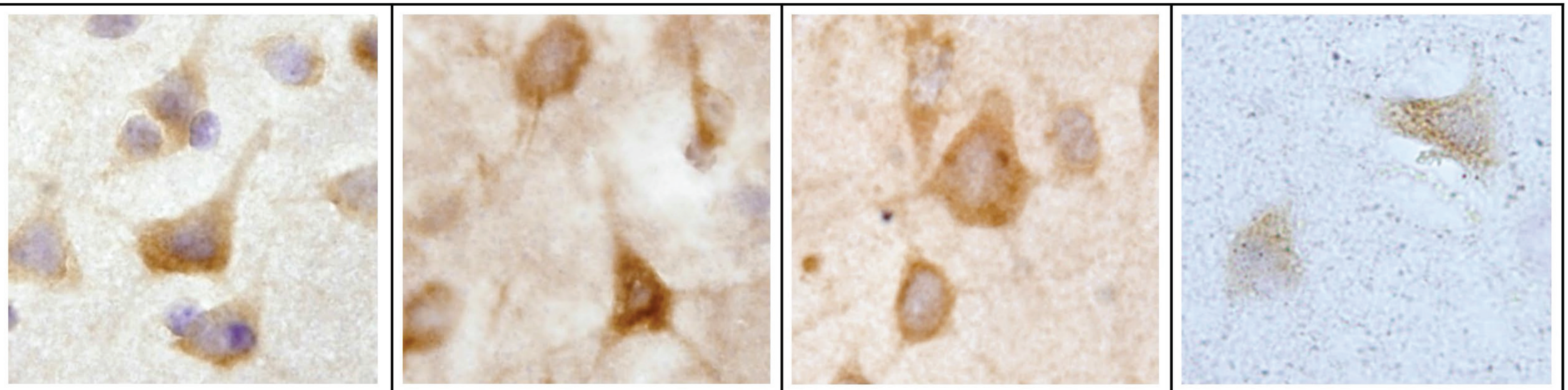

**Fixiation / preservation method**			
4% PFA-fixed, frozen floating			formalin-fixed, paraffin-embedded.
**Heating preparation**			
No preparation	Autoclaving (121℃, 20 min) in Citrate buffer, pH 6	Pressure cooking (120℃, 10 min) in Tris-EDTA buffer, pH 9	
