# Supplementary figures and images for: Intracellular dynamics of Ataxin-2 in the human brains with normal and frontotemporal lobar degeneration with TDP-43 inclusions
Source: Acta Neuropathol Commun. 2020 Oct 28;8:176. doi: 10.1186/s40478-020-01055-9 (PMC7594343; doi:10.1186/s40478-020-01055-9)

Additional file 2: Supplementary Fig. 2

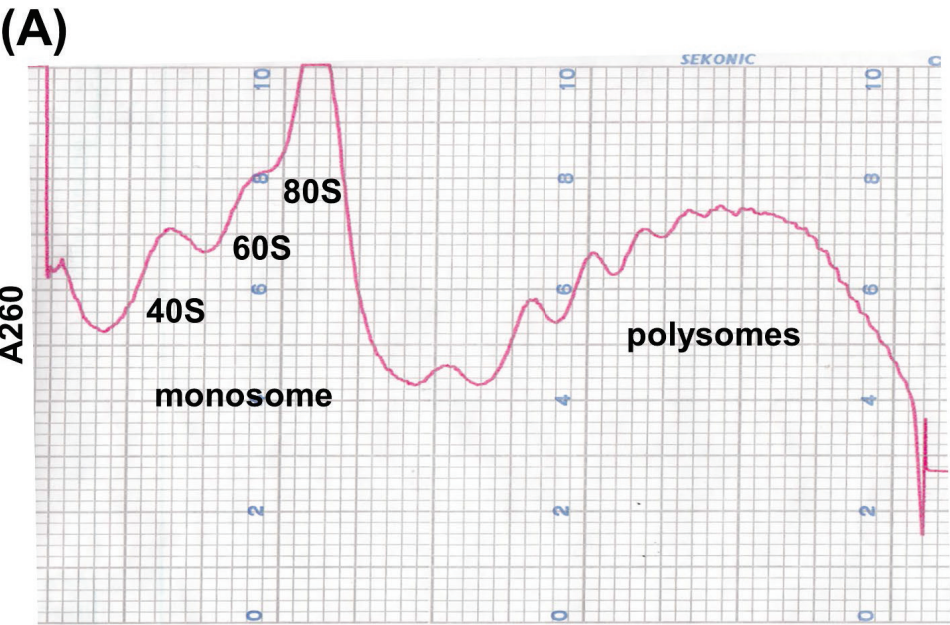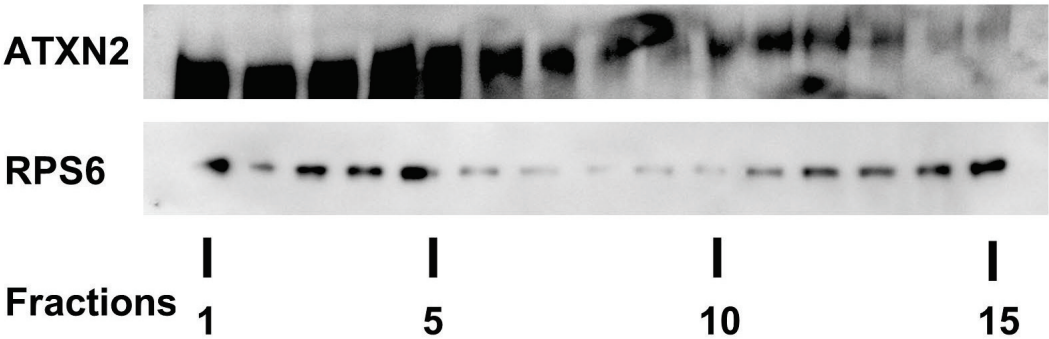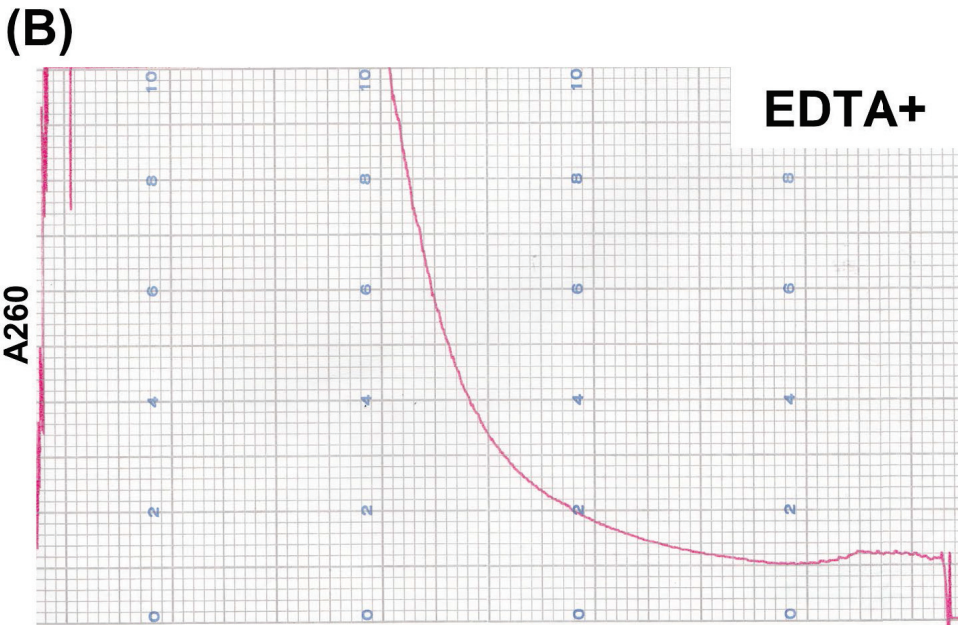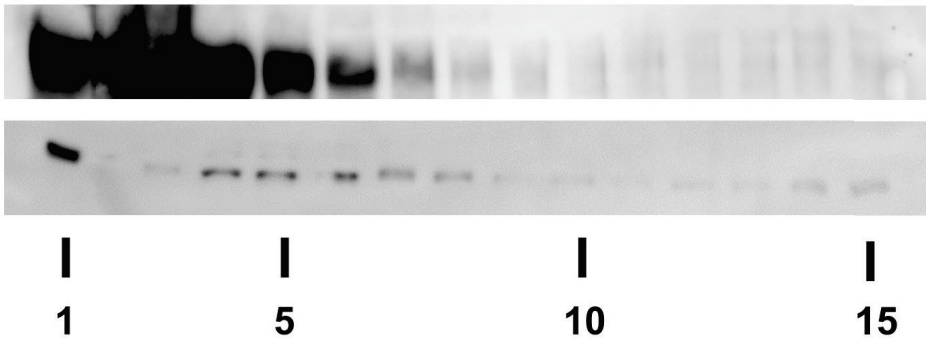

Supplement: Supplementary file 2 — Polysome profiling of ATXN2 in SH-SY5Y lysates. The cell lysates were fractionated in 10–50% (w/v) sucrose density-gradient, collected with a monitoring RNA absorption curve at 260 nm (A260), and analyzed by western blotting. a The analysis of cell lysate without EDTA showed sedimentation of ATXN2 and ribosomal subunit RPS6 in both monosomal and polysomal fractions. b EDTA treatment disrupted the sedimentation of ATXN2 and RPS6 in polysomal fractions. The cropped blots were presented for clarity and conciseness. [file 40478_2020_1055_MOESM2_ESM.pdf]

Additional file 3: Supplementary Fig. 3

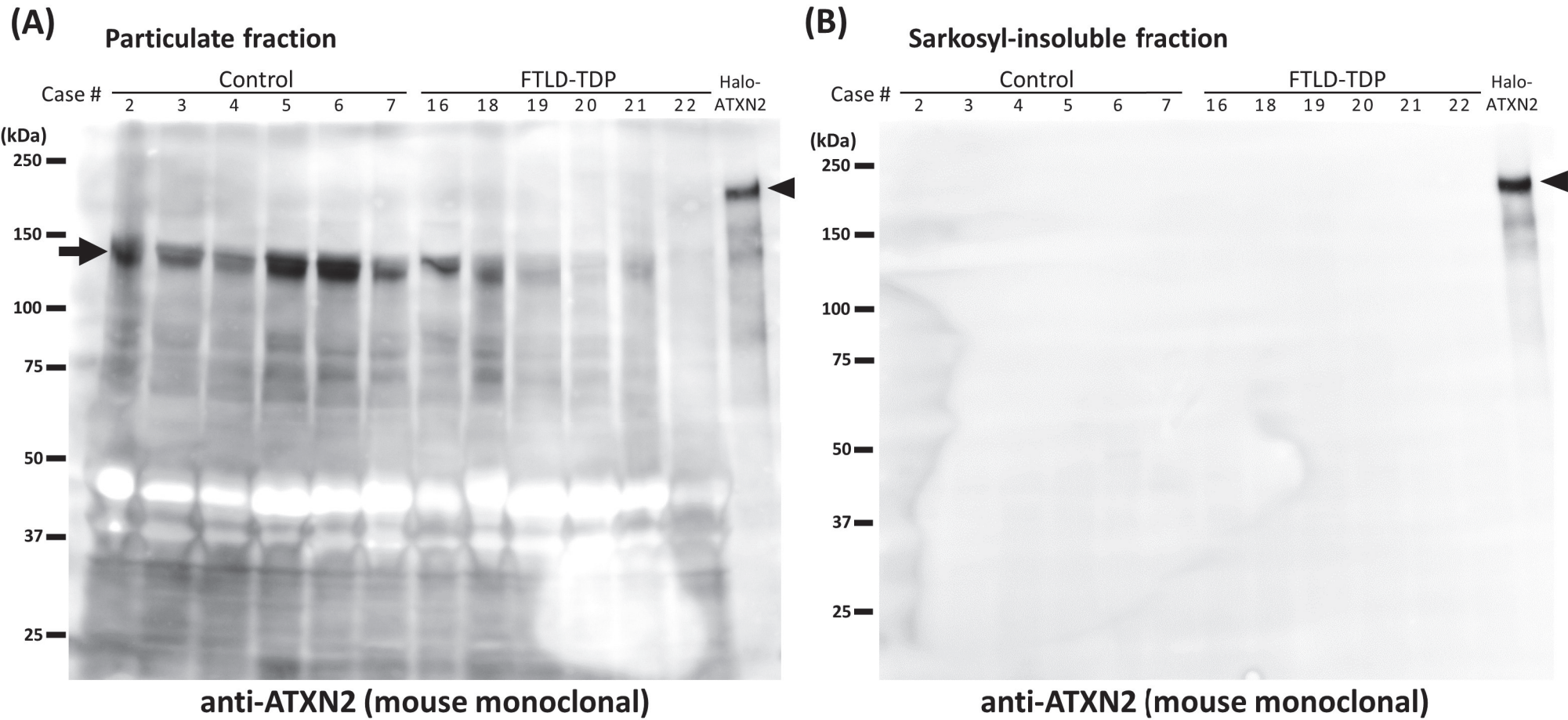

Supplement: Supplementary file 3 — Western blotting analysis of human brain ATXN2. Particulate fractions and sarkosyl-insoluble fractions of brain homogenates from six normal controls and six FTLD-TDP cases were analyzed by western blotting using the mouse monoclonal anti-ATXN2 antibody. Halo-tagged ATXN2 sample was also analyzed as a positive control in each blot (arrowhead). a Immunoblot of particulate fraction. ATXN2 was electrophoresed at approximately 150 kDa (arrow). b Immunoblot of sarkosyl-insoluble fraction. Insoluble ATXN2 was not found in both normal controls and FTLD-TDP cases. [file 40478_2020_1055_MOESM3_ESM.pdf]
